# Ultrafast triggering of strong coupling in a semiconductor terahertz Fabry-Pérot cavity

**DOI:** 10.1038/s41377-026-02379-2

**Published:** 2026-08-19

**Authors:** Soumitra Hazra, Hanna Turchinsky, Tal Schwartz, Sharly Fleischer

**Affiliations:** 1 Raymond and Beverly Sackler Faculty of Exact Sciences, School of Chemistry, Tel Aviv University, Tel Aviv, 6997801 Israel; 2 Tel Aviv University Center for Light-Matter Interaction, Tel Aviv, 6997801 Israel

**Keywords:** Photonic devices, Terahertz optics, Polaritons, Ultrafast photonics

## Abstract

Strong light-matter coupling – the hybridization of material excitations with the electromagnetic modes of a photonic resonator, has led to striking modifications in the physical and chemical properties of materials, including their chemical reactivity. To date, experimental studies of these effects have been limited to steady-state conditions, namely long after the formation of polaritons. Here, we overcome this constraint by introducing an ultrafast, optically switchable Fabry–Pérot cavity that enables *on-demand* triggering of strong coupling with a switch-on time of just a few picoseconds. We demonstrate dynamic activation of strong coupling in the terahertz domain between a cavity mode and the collective vibration of crystalline *α*-lactose powder pellet embedded in the cavity. By photoexciting silicon-based cavity mirrors, we induce an abrupt spectral shift and linewidth narrowing of the photonic cavity mode alone – leading to a substantial increase in the Q-factor and tuning the cavity mode into resonance with the vibrational transition of the cavity-embedded material. This, in turn, enables ultrafast light-matter hybridization through the formation of vibrational polaritons. The ability to switch the cavity ‘on demand’ at picosecond timescales provides means to actively trigger strong light-matter coupling and to optically manipulate the coupling strength, offering new opportunities to study its dynamics and guide chemical reactivity.

**Figure:**
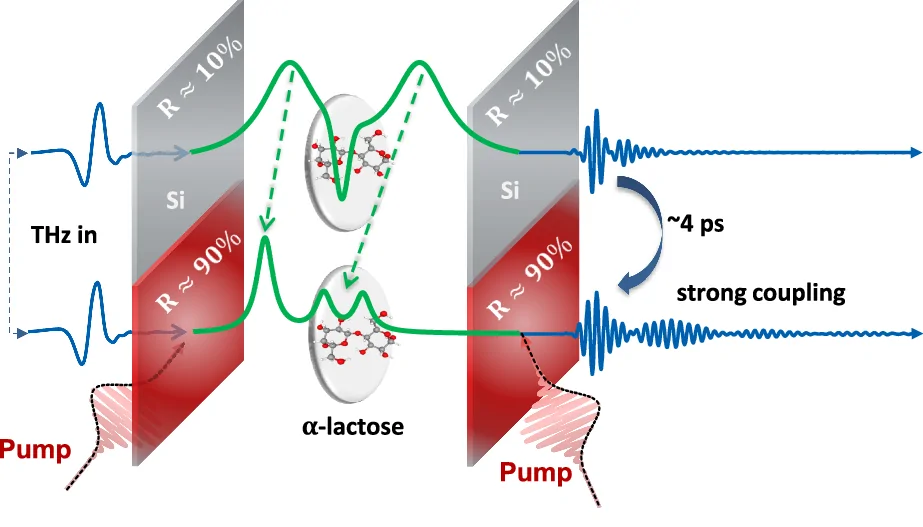

In strong light-matter coupling (SC), the interaction between a material and a photonic mode of an optical resonator results in hybrid polaritonic states shared by the two. Physical and chemical properties of materials were shown to be significantly modified under SC and recently, intriguing modifications in chemical reactions performed under SC conditions, have launched a world-wide pursuit of polaritonic chemistry^1–7^, and reinforced interest in the broader SC phenomena^8–11^.

Over the past decades, SC has been explored and demonstrated in various photonic cavity platforms such as distributed Bragg reflector microcavities^12–15^, photonic^16–18^ and plasmonic nanocavities^19^ and metasurfaces^20^, metallic cavities^21,22^, photonic crystal cavities^23^ and other configurations. Among the physical and chemical properties that have shown to significantly modify under SC are charge transport^24–26^ and energy transport^27,28^, non-linear susceptibility^29^ and polarizability^30^, metal-insulator transitions^31^, topological protection^32^, selective crystallization of metal-organic frameworks^33^ and the rate of molecular chemical reactions^1,3,34–36^.

The underlying physics of SC is often described in the framework of quantum electrodynamics (QED), involving vacuum fluctuations that emerge within the cavity environment^37–40^. As such, polaritonic chemistry experiments do not require any external photon source and are often realized ‘in the dark’. In-situ spectroscopic measurements, commonly employed to monitor SC conditions and reaction progression, do not interfere with polariton formation, nor do they alter the still-unexplained outcomes of polaritonic chemistry. In light of this scientific puzzle, a natural approach has been to diversify experimental scenarios and conditions. To this end, SC has been modulated through various schemes including external gate voltage^41–43^, tip-enhanced SC in the nanoscale^44^, acoustic shockwaves^45^, electro-luminescence^46^ and photo-doping^22^ among others. Ultrafast control of SC on femtosecond (fs) timescales has been demonstrated in cavity-embedded quantum wells^47–49^ and by ultrafast excitation of the cavity-embedded material^50^ and down to sub-cycle switching intersubband transitions within a semiconductor microcavity^49,51^. However, a key capability remains elusive: the ability to switch SC ‘on demand’ and noninvasively — that is, without perturbing the molecular system itself. Such control is crucial for isolating the causal role of strong coupling in chemical dynamics and for directly probing its mechanistic impact on reaction pathways. In this work, we present a platform that delivers precisely this functionality.

We present and demonstrate a new, versatile approach for ultrafast tuning of the photonic cavity mode into resonance with the transition of the embedded material that, as will be shown, triggers SC and manifest by the formation of polaritons. With polaritonic chemistry applications in mind, we require a control scheme that modifies only the cavity properties, without directly affecting the embedded material; i.e. the quantum states distribution and number of transition dipoles must remain unaffected. This control scheme is implemented in a Fabry-Pérot (FP) cavity formed by thin silicon (Si) semiconductor mirrors, which are photoexcited (PE) by above band-gap ($800\,{\rm{nm}}$) ultrashort ($\sim 110\,{\rm{fs}}$) pulses. The resulting carrier injection produces an abrupt and long-lived enhancement of the cavity Q-factor and a shift of the cavity resonance frequency, without directly affecting the embedded material. We implement this scheme to optically control the strong coupling between a terahertz (THz) cavity and the collective vibrational transition of α-Lactose Monohydrate (*α*-LM) crystallites^52^, and demonstrate a switching time of $4\,{\rm{ps}}$, which is much shorter than the photon life-time of the cavity.

This paper is organized as follows: first we present the experimental setup utilized for this work and characterize the THz properties of a single ultrathin Si mirror upon PE by the control pulse. Next we present the extent of control over an empty FP cavity constructed from two Si mirrors. In the last section we experimentally validate the presented technique by demonstrating ultrafast triggering of SC with *α*-LM embedded within the switchable FP cavity.

## Experimental setup

### Dual near-IR pump - THz time-domain spectrometer

**Fig. 1:**
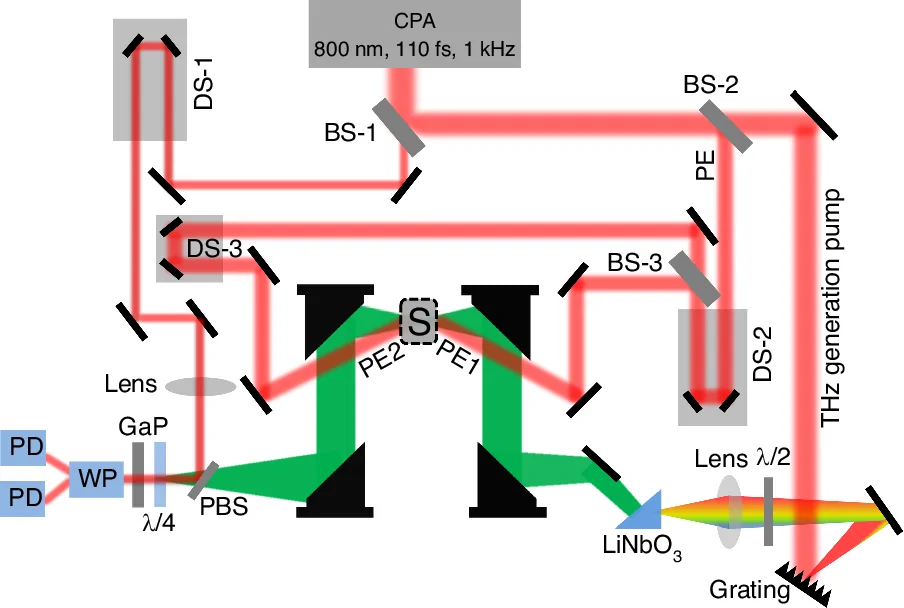
Experimental setup – Time domain THz spectrometer equipped with two photoexcitation pulses (PE1, PE2) for sample (S) excitation. The single-cycle THz pulse is generated via the tilted pulse-front optical rectification in ${\rm{LiNb}}{{\rm{O}}}_{3}$ prism-cut crystal and detected by electro-optics sampling in a GaP crystal. The timing of all beams with respect to the THz is provided by linear translation stages (DS-1, DS-2, DS-3). BS - beam splitter, PBS –pellicle beam splitter, WP – Wollaston prism, PD - photodiode. Throughout the paper we refer to the PE pulse energy$[{\rm{\mu }}{\rm{J}}]$. Converting between PE pulse energy to PE fluence affecting the THz probe transmission is given by: 100 μJ↔120 μJ cm^-2^

The experiments were performed using a home-built dual near-IR pump – THz probe time-domain spectrometer, as shown in Fig. 1. An ultrashort ($110\,{\rm{fs}},\,800\,{\rm{nm}}$) pulse from a Ti: Sapphire chirped pulse amplifier ($1{\rm{KHz}}$ repetition rate) was split in three: $\sim 900\,{\rm{\mu }}{\rm{J}}$ was used for generating the THz field, $\sim 450\,{\rm{\mu }}{\rm{J}}$ for PE of the Si mirrors and a weak $\sim 1\,{\rm{\mu }}{\rm{J}}$ read-out pulse for electro-optic THz detection in a $1\,{\rm{mm}}$ thick GaP crystal^53,54^. Single-cycle THz pulses were generated using tilted pulse-front optical rectification^55^ with $0.1-1.2\,{\rm{THz}}$ usable bandwidth. The PE beam was routed through a computer-controlled delay stage and split in two (marked PE1, PE2 in Fig. 1) for PE simultaneously the two Si wafers. The THz beam diameter at the sample position (S) was focused to $4\,{\rm{mm}}$, at the center of the PE1 (and PE2) beam. The delays of the two PE pulses with respect to the THz pulse and their pulse energies were set equal using an additional delay stage and two attenuators (which are not shown in Fig. 1).

## Results

### THz characterization of a photo-excited thin Si wafer

When a semiconductor is excited above band-gap, its optical properties are altered as it switches to the metallic state^56^. For an undoped Si wafer with few hundreds ${\rm{\mu }}{\rm{m}}$ thickness, PE by an intense fs, $800\,{\rm{nm}}$ pulse results in an abrupt enhancement of THz reflectivity, from $\sim 50 \%$ to >90%^57^, which persists for a few ${\rm{ns}}$ until recombination is completed^58^. However, for a sufficiently thin (sub-wavelength) Si substrate, used in this work as switchable cavity mirrors, the THz reflectivity of the unexcited Si is $\sim 10 \%$ (and $\sim 90 \%$ THz transmission) owing to the zero-order Fabry-Pérot mode of the thin wafer^59^.

We start by characterizing the THz properties of a single Si mirror with thickness ${\rm{d}}=12\,{\rm{\mu }}{\rm{m}}$ subjected to PE by short $110{\rm{fs}}$, Near-IR ($800\,{\rm{nm}}$) pulse^60^. The THz transmission of the unexcited, free-standing Si mirror, placed at the sample position (marked ‘S’ in Fig. 1) was measured (with beam ‘PE1’ while ‘PE2’ is blocked) and its complex refractive index $(\widetilde{n}=n+{ik})$ extracted by minimizing the difference between the experimental and theoretical transmission function^61^:

1 $$\widetilde{T}\left(\omega \right)=\frac{{\widetilde{E}}_{{sample}}\left(\omega \right)}{{\widetilde{E}}_{{air}}\left(\omega \right)}=\frac{4\widetilde{n}}{{\left(\widetilde{n}+1\right)}^{2}}\cdot \exp \left[i\frac{\left(\widetilde{n}-1\right)\omega d}{c}\right]\cdot \frac{1}{1-{\left(\frac{\tilde{n}-1}{\tilde{n}+1}\right)}^{2}\cdot \,{e}^{\frac{i2\tilde{n}\omega d}{c}}}$$

The numerical procedure for extracting $\widetilde{n}$ is detailed in SI section S1.

**Fig. 2:**
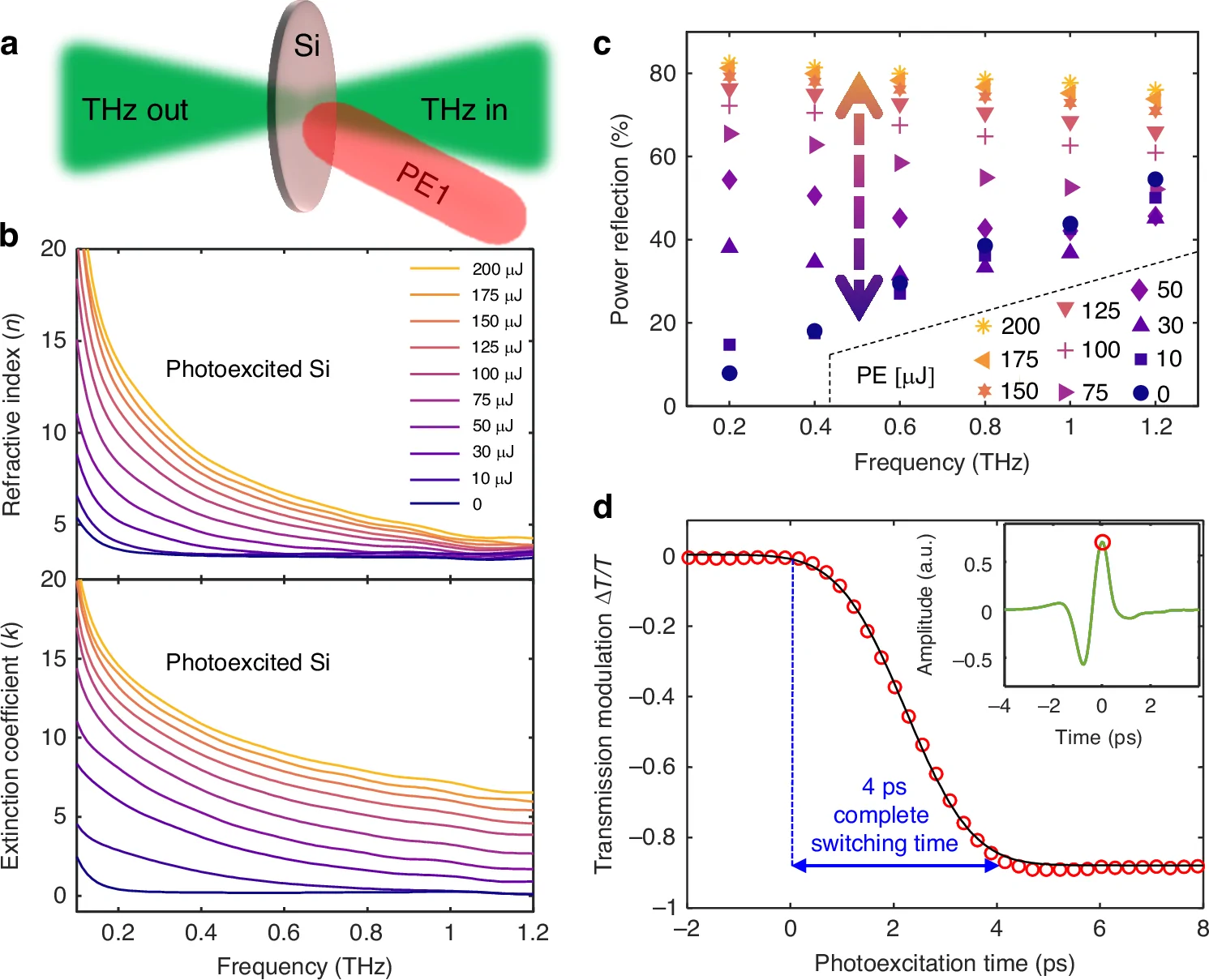
THz characterization of PE 12 μm Si wafer. **a** A scheme of THz transmission through a single Si mirror, excited by the near-IR pulse (PE1). **b** Experimentally extracted refractive index $\left(n\right)$ and extinction coefficient (k) for varying PE1 energies. **c** Calculated power reflection as a function of PE1 energy (see legend) for selective THz frequencies. The double-sided arrow marks the $0.53\,{\rm{THz}}$ region of interest, where PE1-induced reflectivity ranges from ~20–80% (**d**) Modulation of THz peak transmission (shown in the inset) vs. PE1 delay. Red circles mark the modulation of the peak THz field (shown in the inset) obtained in the experiment. The black curve is a fitted by an error function $\frac{\varDelta T}{T}=A[1-{erf}\left(\frac{t-{t}_{0}}{\sqrt{2}\sigma }\right)]$ yielding a 10% - 90% of modulation range switching time of $2.6\,{\rm{ps}}$. The complete switching time of $4\,{\rm{ps}}$ is marked by the blue double-sided arrow

Next, we characterized $\widetilde{n}$ of the Si wafer following PE by beam ‘PE1’ (while ‘PE2’ is blocked, Fig. 2a) at varying pulse energies using the same procedure. [Note: throughout the paper $100\,{\rm{\mu }}{\rm{J}}$ PE energy corresponds to $120\,{\rm{\mu }}{\rm{J}}\,{{\rm{cm}}}^{-2}$ PE fluence]. Since the thickness of our Si wafer $(12\,{\rm{\mu }}{\rm{m}})$ is smaller than the penetration depth of undoped Si at $800\,{\rm{nm}}$ ($\sim 13.5\,{\rm{\mu }}{\rm{m}}$, ref.^62^), we assume homogeneous PE and uniform effective index.

Figure 2b depicts the real (n) and imaginary (k) parts of the refractive index of the PE Si (Si^*^) in the frequency range of our THz field ($0.2-1.2\,{\rm{THz}}$), showing gradual increase in both n and k with PE1 pulse energy (for the complex permittivity of Si^*^ see SI section S7). Figure 2c depicts the calculated power reflectance of Si^*^at several PE1 energies, with a $\sim 10 \% \to 85 \%$ modulation range for the low THz frequencies and $\sim 40 \% \to 80 \%$ for higher frequencies, showing the tunability range of the ultrathin Si mirrors provided by $800\,{\rm{nm}}$ PE (see SI section S2). The temporal response (‘switching time’) of the Si was measured by parking the EO readout pulse at the peak of the transmitted THz transient (marked in the inset of Fig. 2d) as a function of PE1 delay. The peak THz transmission drops by a factor of 10 within $4\,{\rm{ps}}$ after photoexcitation, establishing an upper bound for complete switching (blue arrow in Fig. 2d), and is attributed to thermalization of hot carriers via scattering and phonon interactions in Si.^63^

### Switchable Fabry-Pérot Si cavity

**Fig. 3:**
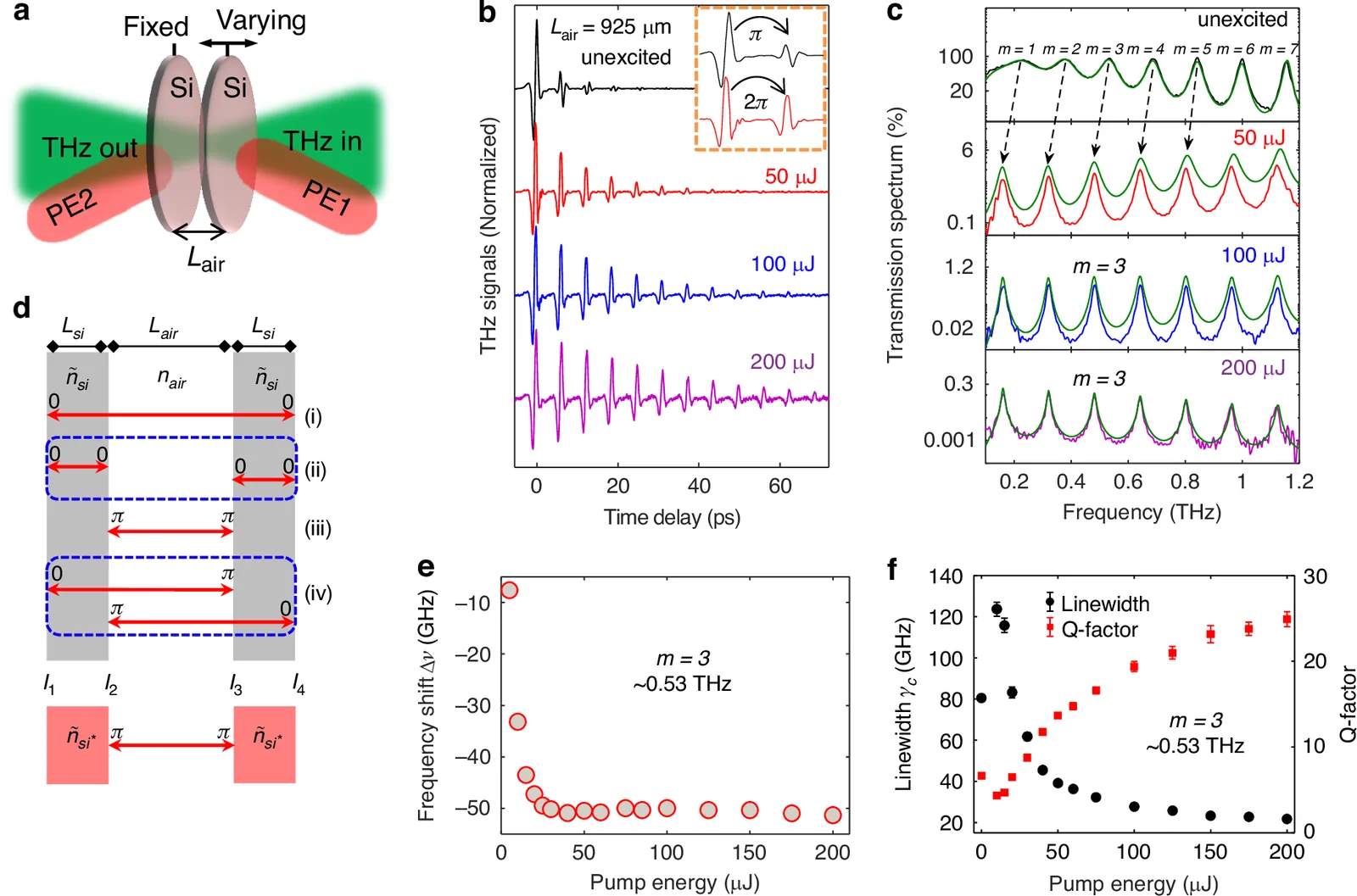
Characterization of the switchable Fabry-Pérot Si cavity. **a** Measurement scheme: empty FP cavity made of two Si wafers excited simultaneously by two near-IR pulses (PE1, PE2). In all of the measurements the THz probe succeeded the PE pulses by $7\,{\rm{ps}}$ and ${L}_{{air}}$ was kept fixed at $925\,{\rm{\mu }}{\rm{m}}$ (**b**) Time-resolved THz transmission of unexcited and PE cavity at different pulse energies (color-coded). The inset shows the phase shift between adjacent transients of the unexcited cavity (black curve) and PE cavity (red curve). **c** Transmission spectra obtained by Fourier transformation of the TD-THz signals of Fig. 3b, showing multiple FP modes. The PE-induced redshifts of the modes are marked by dashed black arrows. TMM calculations are shown by solid green lines. **d** A sketch of the FP cavity for unexcited (grey) cavity and PE (red) cavity. Possible mode configurations are marked by double-sided red arrows, and the reflection phase at each interface is noted. Degenerate modes are grouped by the dashed blue rectangles. **e** The detuning of the m=3 cavity mode (set $\sim 0.53\,{\rm{THz}}$) as a function of PE energy. **f** The linewidth ${(\gamma }_{c})$ and cavity Q-factor of the m=3 mode at varying PE energies

Next we characterize the PE-induced response of an empty Fabry-Pérot cavity constructed of two free-standing Si wafers situated parallel to each other (Fig. 3a). One of the Si wafers was placed on a translation stage for precise control of the cavity length $({L}_{{air}})$.

Figure 3b shows the time resolved THz transmission of the cavity with ${L}_{{air}}=925\,{\rm{\mu }}{\rm{m}}$ for the unexcited (black curve) and excited (by both PE1 and PE2) with $50\,{\rm{\mu }}{\rm{J}},100\,{\rm{\mu }}{\rm{J}}$ and $200\,{\rm{\mu }}{\rm{J}}$ (Red, Blue and Pink respectively, each normalized by the first transient). A series of pulses (cavity reflections), separated by the cavity roundtrip is observed. In the unexcited cavity this series of reflections is strongly damped due to the low reflectivity of the Si wafers (low-Q cavity, short lifetime). Upon PE by PE1 and PE2, the Si wafers turn into effective cavity mirrors, manifested by increased cavity lifetime (reduced damping, higher-Q) as PE energy increases. We note that the PE pulses preceded the THz probe by $7\,{\rm{ps}}$ to accommodate for the $4\,{\rm{ps}}$ switching time and the complete duration of the THz field ($\sim 3\,{\rm{ps}}$, see inset in Fig. 2d). For longer delays between the PE and THz up to $\sim 300\,{\rm{ps}}$ (the maximal delay afforded by our setup), the transmission spectra remains identical to that presented (with $7\,{\rm{ps}}$). Figure 3c shows the power spectrum obtained by Fourier transformation of the time-domain signals of Fig. 3b. The solid green curves, calculated using the transfer matrix method (TMM; see Methods section) are in good agreement with color-coded experimental results. In the frequency domain, the increased lifetime of the PE cavity manifests as a narrowing of the FP mode linewidths with increasing PE energy.

A key finding of Fig. 3c is the redshift of the FP modes upon PE of the Si wafers (marked by the dashed black arrows), where the *m* = 1 cavity mode shifted from $0.23\,{\rm{THz}}\to 0.16\,{\rm{THz}}$ upon PE, $m=2:\,0.38\,{\rm{THz}}\to 0.32\,{\rm{THz}}$, $m=3:\,0.53\,{\rm{THz}}\to 0.48\,{\rm{THz}}$ with the same trend at higher modes. To explain the red-detuning of the cavity upon PE, we must first identify the effective cavity boundaries (interfaces) of the unexcited cavity modes.

### Identification of the dominant unexcited cavity mode

The unexcited Si–air–Si stack supports several possible mode configurations (Fig. 3d, i-iv). Comparison between analytical transfer-function calculations (for the selective mode configurations shown in Fig. 3d) and TMM calculation of the entire Si-air-Si stack, identify configuration (iv), degenerate modes bounded by the inner air–Si interface of one mirror and the outer Si–air interface of the other, as the dominant contribution to the unexcited cavity spectrum. This is confirmed by the time-domain signal (Fig. 3b, inset), where consecutive THz transients alternate in phase by ${\rm{\pi }}$. Such a shift is a direct manifestation of Fresnel reflection, requiring one reflection at a low-to-high index interface (air-Si) and one at a high-to-low interface (Si-air) per roundtrip. For a detailed derivation and selective analytical calculations see SI Section S9.

### Mechanism of the PE-induced redshift

Upon photoexcitation (PE), the Si wafers transform into effective metallic mirrors (Si*), confining the THz field between the two inner air–Si* interfaces (see excited cavity colored red in Fig. 3d). This transition induces two competing effects on the resonance frequency, governed by the Fabry-Pérot (FP) round-trip phase condition,

2 $${\phi }_{{rt}}={\phi }_{{prop}}+{\phi }_{r1}+{\phi }_{r2}=2{\rm{\pi }}q$$

(where ${\phi }_{{prop}},\,{\phi }_{r1},\,{\phi }_{r2}$ are the propagation and reflection phases at the two cavity interfaces respectively and q is an integer).

*Effect 1 - Reflection Phase Shift:*

- Unexcited Cavity: Based on the identified dominant mode (configuration iv), the field accumulates a total reflection phase of ${\rm{\pi }}$ per round trip (${\phi }_{r1}\, \sim \,0$ at Si-air and ${\phi }_{r2}\, \sim \,{\rm{\pi }}$ at air-Si).
- Excited Cavity: After PE, the field is confined by two metallic Si* mirrors, each contributing a ${\rm{\pi }}$ phase shift. The total round-trip reflection phase thus doubles to $2{\rm{\pi }}$.

*Effect 2 - Physical Shortening*: The reflecting planes shift from the outer Si-air interfaces to the inner air-Si* interfaces. This reduces the propagation length by ${n}_{{Si}}\cdot {L}_{{Si}}$ per mirror, which by itself would cause a blueshift.

### Resonance frequencies of the two states of the cavity

Substituting the respective reflection phases at the cavity interfaces and optical paths into the resonance condition of Eq.2 and defining m≡q−1 as mode number (See SI section 8), we derive the resonance frequencies $(\nu =c/\lambda )$ for the two states of the cavity:

For the unexcited cavity:

$$\frac{4\pi ({n}_{{Si}}{\cdot L}_{{Si}}+{n}_{{air}}\cdot {L}_{{air}})}{{\lambda }_{{un}}}+\mathop{\underbrace{{\phi }_{{Si},{air}}}}\limits_{\approx 0}+\mathop{\underbrace{{\phi }_{{air},{Si}}}}\limits_{\approx \pi }=2\pi (m+1)\Longrightarrow {{\boldsymbol{\nu }}}_{{\boldsymbol{un}}}=\frac{({\bf{2}}{\boldsymbol{m}}{\boldsymbol{+}}{\bf{1}})\cdot {\boldsymbol{c}}}{{\bf{4}}\cdot ({{\boldsymbol{n}}}_{{\boldsymbol{Si}}}{\cdot {\boldsymbol{L}}}_{{\boldsymbol{Si}}}{\boldsymbol{+}}{{\boldsymbol{n}}}_{{\boldsymbol{air}}}{\cdot {\boldsymbol{L}}}_{{\boldsymbol{air}}})}$$

For the excited cavity:

$$\frac{4\pi ({n}_{{air}}\cdot {L}_{{air}})}{{\lambda }_{{ex}}}+\mathop{\underbrace{{\phi }_{{air},{{Si}}^{* }}}}\limits_{=\pi }+\mathop{\underbrace{{\phi }_{{air},{{Si}}^{* }}}}\limits_{=\pi }=2\pi (m+1)\Longrightarrow {{\boldsymbol{\nu }}}_{{\boldsymbol{ex}}}=\frac{{\bf{2}}{\boldsymbol{m}}\cdot {\boldsymbol{c}}}{{\bf{4}}{\cdot {\boldsymbol{n}}}_{{\boldsymbol{air}}}{\cdot {\boldsymbol{L}}}_{{\boldsymbol{air}}}}$$

where ${\phi }_{i,j}$ are the reflection phases at the cavity interfaces^64^ where ${\phi }_{i,j}={{\arg }}\left({\widetilde{r}}_{i,j}\right)$ and ${\widetilde{r}}_{i,j}=\frac{{\tilde{n}}_{i}-{\tilde{n}}_{j}}{{\tilde{n}}_{i}+{\tilde{n}}_{j}}$ is the reflection coefficient (SI section S3).

For our experimental parameters (${L}_{{Si}}=12\,{\rm{\mu }}{\rm{m}},\,{n}_{{Si}}\cong 3.42,\,{L}_{{air}}=925\,{\rm{\mu }}{\rm{m}},\,{n}_{{air}}=1,\,m=3$), the calculated values, ${\nu }_{{un}}=0.54\,{\rm{THz}}$ and ${\nu }_{{ex}}=0.48\,{\rm{THz}}$ are in excellent agreement with the experimental results and TMM simulations. This confirms that the additional ${\rm{\pi }}$ reflection phase increases the effective optical length by $\sim \lambda /2$, overriding the ${n}_{{Si}}{\cdot L}_{{Si}} \sim 41\,{\rm{\mu }}{\rm{m}}$ physical shortening and resulting in the observed redshift.

While the above analytical expressions for ${\nu }_{{un}}$ and ${\nu }_{{ex}}$ neglect THz dispersion in Si and approximate reflection phases as either 0 or π for brevity, they accurately reproduce the resonance frequencies shown in Fig. 3c. Beyond this validation, the model provides a framework to design switchable cavities tailored to specific material transition frequencies by predicting the resulting PE-induced shifts (see SI sections S4 and S11 for further examples and design tables).

### PE energy – dependent cavity response

To further characterize the switchable response, we examine an empty cavity $({L}_{{air}}=836\,{\rm{\mu }}{\rm{m}})$, where the unexcited m=3 mode is ${\nu }_{{un}}\approx 0.58\,{\rm{THz}}$. Figure 3e shows the experimentally measured detuning $(\triangle \nu ={\nu }_{{ex}}-{\nu }_{{un}})$ as a function of PE energy. The mode gradually red-detunes with increasing energy, reaching a stable shift of $\triangle \nu \,\simeq -50\,{\rm{GHz}}$ at $35\,{\rm{\mu }}{\rm{J}}$ – a limit bound by the π reflection phase at the air-Si^*^ interface. Simultaneously, the cavity Q-factor increases from ~6.5 (unexcited) to ~25 at $200\,{\rm{\mu }}{\rm{J}}$ as the Si^*^ mirrors become increasingly reflective (Fig. 3f). At low PE energies $(0\,{\rm{\mu }}{\rm{J}}\to 20\,{\rm{\mu }}{\rm{J}})$ the Si has not yet reached a fully metallic state; in this intermediate regime, increased absorption (see Fig. 2b for extinction coefficient) causes a transient broadening of the linewidth (${\gamma }_{c}$ increases from $80\,{\rm{GHz}}$ to $123\,{\rm{GHz}}$) and a slight reduction (from 6.5 to ~5) in Q-factor (see SI section S7).

### **Ultrafast triggered strong-coupling of*α*-lactose monohydrate in PE Si cavity**

**Fig. 4:**
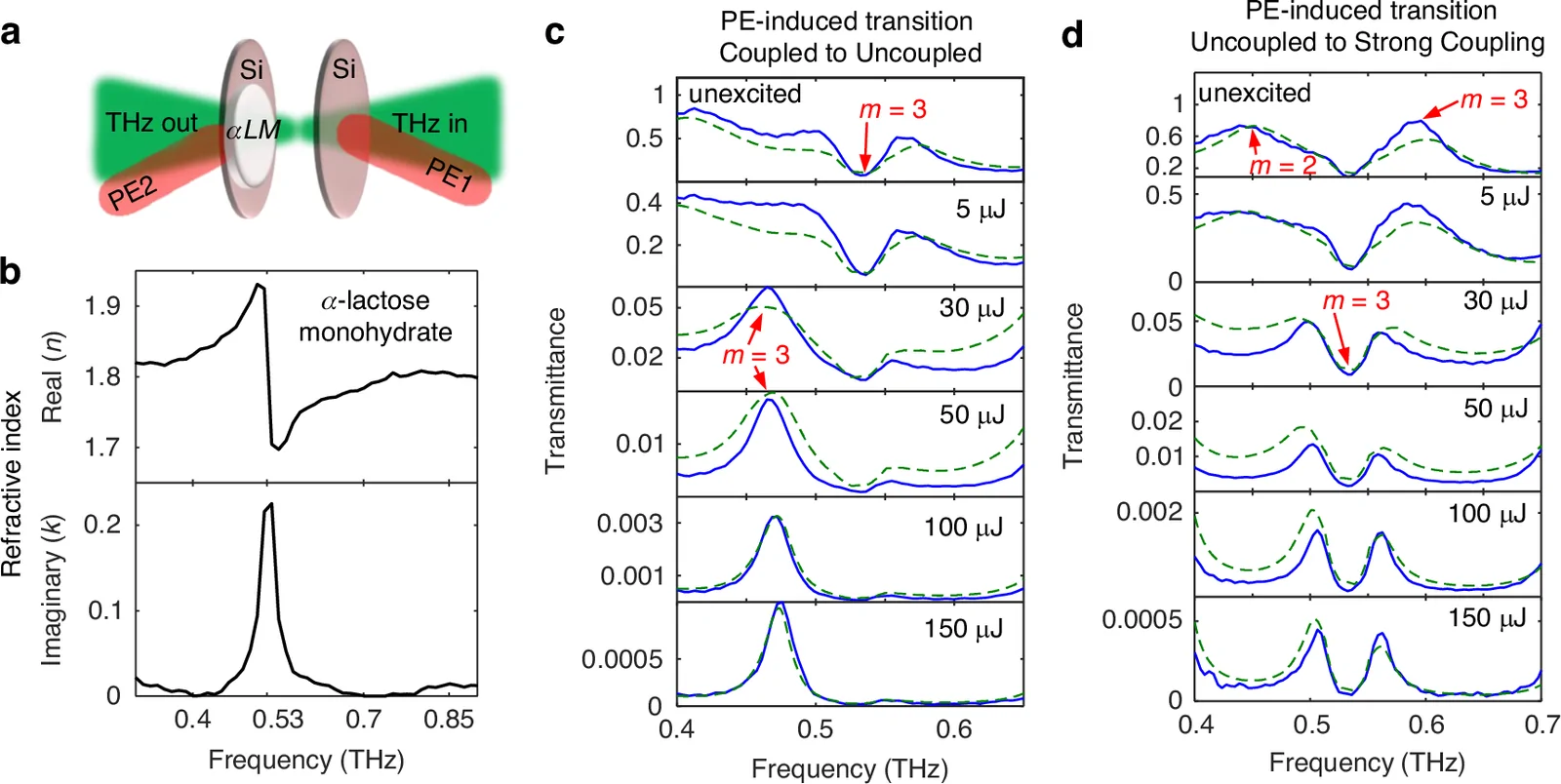
Triggering strong coupling of α-lactose in cavity. **a** Measurement scheme: switchable FP cavity with a 270$\,{\rm{\mu }}{\rm{m}}$ α-LM pellet embedded inside. **b** Complex refractive index of α-LM extracted from time-domain THz spectroscopy of a free-standing α-LM pellet. **c** PE-induced decoupling of cavity and α-LM showing a transition from the coupled state (upper panel, unexcited) to the uncoupled state (lower panel, $150\,{\rm{\mu }}{\rm{J}}$). **d** PE-induced strong-coupling of cavity and α-LM showing a transition from the uncoupled state (upper panel, unexcited) to the strongly coupled state (lower panel,$\,150\,{\rm{\mu }}{\rm{J}}$). THz transmission spectra with varying PE pulse energies are shown. Blue curves in (**c**) and (**d**) depict the experimental data overlaid with their corresponding TMM simulations depicted by the dashed green curves. In both scenarios the PE pulse preceded the THz probe by $7\,{\rm{ps}}$ to accommodate the $4\,{\rm{ps}}$ switching time and the temporal width ($\sim 3\,{\rm{ps}}$) of the THz

In what follows we utilize the switchable FP Si cavity to trigger strong-coupling between *α*-lactose monohydrate (*α*-LM) and PE Si cavity. Limited by the fragility of *α*-LM pellets with thickness below $\sim 100\,{\rm{\mu }}{\rm{m}}$ and in order to secure sufficient air-gap for cavity detuning measurements, we chose to couple the collective vibration of *α*-LM at $0.53\,{\rm{THz}}$^52^ with the third mode of the cavity (*m* = 3). *α*-LM crystallites in powder form (Sigma-Aldrich CAS No: 5989-81-1) were pressed in a pressing die to form a $270\,{\rm{\mu }}{\rm{m}}$ thick pellet sample (see methods section). The complex refractive index of the free-standing *α*-LM pellet was extracted from THz transmission measurements and is shown in Fig. 4b. Next, the *α*-LM pellet was mounted on one of the Si wafers and the open FP cavity was constructed by placing another Si wafer parallel to the first (Fig. 4a). The latter was mounted on a computer-controlled linear translation stage to enable precise cavity detuning measurements.

Figure 4c, d feature the main experimental scenarios provided by the switchable cavity: PE-induced light-matter decoupling (4c) and PE-induced strong light-matter coupling (4d). In both scenarios (Fig. 4c, d) the PE pulse preceded the THz probe by $7\,{\rm{ps}}$ beyond which (up to $\sim 300\,{\rm{ps}}$, limited by our delay stage), the transmission spectra remain identical to that obtained with $7{\rm{ps}}$.

In Fig. 4c we set ${L}_{{air}}=410\,{\rm{\mu }}{\rm{m}}$ such that the *m* = 3 frequency of the unexcited cavity is resonant with the $0.53\,{\rm{THz}}$ transition of *α*-LM. The transmission spectrum in the range $0.4\,{\rm{THz}}-0.65\,{\rm{THz}}$ is depicted for several PE energies. The unexcited cavity (upper panel of Fig. 4c) shows a frequency splitting of $67\,{\rm{GHz}}$ around $0.53\,{\rm{THz}}$. Upon PE of the Si wafers with $5\,{\rm{\mu }}{\rm{J}}$ (PE1 and PE2), the mode undergoes a slight redshift, resulting in a small detuning of the coupled system from resonance. With $30\,{\rm{\mu }}{\rm{J}}$ the m=3 mode is redshifted by $\sim 50\,{\rm{GHz}}$, and is far-detuned from the *α*-LM transition. Further increase in PE energy from $30-150\,{\rm{\mu }}{\rm{J}}$ results in gradual narrowing (Q-factor increase) albeit without further detuning, in agreement with the data of Fig. 3e, f. We note that the coupling strength of the unexcited Si cavity is significantly limited by the low reflectivity of unexcited Si. Nevertheless, the potential for all optical PE-based control of the cavity-material coupling is clearly demonstrated.

In the remaining of the paper we analyze the scenario of Fig. 4d where the switchable cavity is utilized as a dynamic mechanism to abruptly transition the system from completely uncoupled state to the strong light-matter coupling regime.

The upper panel of Fig. 4d shows the transmission spectrum of the unexcited cavity with ${L}_{{air}}=320\,{\rm{\mu }}{\rm{m}}$, such that both the m=2 ($0.45\,{\rm{THz}}$) and m=3 $(0.58\,{\rm{THz}})$ modes (marked by red arrows) are far detuned from the *α*-LM transition frequency which manifest as an absorption dip at $0.53\,{\rm{THz}}$. Upon weak PE with $5\,{\rm{\mu }}{\rm{J}}$, the FP modes are slightly redshifted and remain far-detuned from the *α*-LM transition. With $30\,{\rm{\mu }}{\rm{J}}$ PE energy, the m=3 mode is redshifted to $0.53\,{\rm{THz}}$ and strong-coupling is evidenced by polariton splitting (the m=2 mode frequency is shifted to $0.39\,{\rm{THz}}$, beyond the lower limit of the figure). Increasing the PE energy to $150\,{\rm{\mu }}{\rm{J}}$ results in gradual narrowing of the polariton spectra accompanied by a slight reduction in the splitting frequency. In order to account for possible thermal-induced reflectivity bias of the Si wafers that may develop through long-time irradiation by PE1 and PE2 (1 ${\rm{KHz}}$ repetition rate), we reversed the time-ordering of the pulses such that the THz probe precedes the PE by $\sim 60\,{\rm{ps}}$. A negligibly small thermally induced cavity response was observed, with a transmission spectrum practically identical to that of the unexcited cavity (see SI section S6), confirming that the observed cavity response originates solely from above band-gap PE.

## Analysis of the coupling strength

**Fig. 5:**
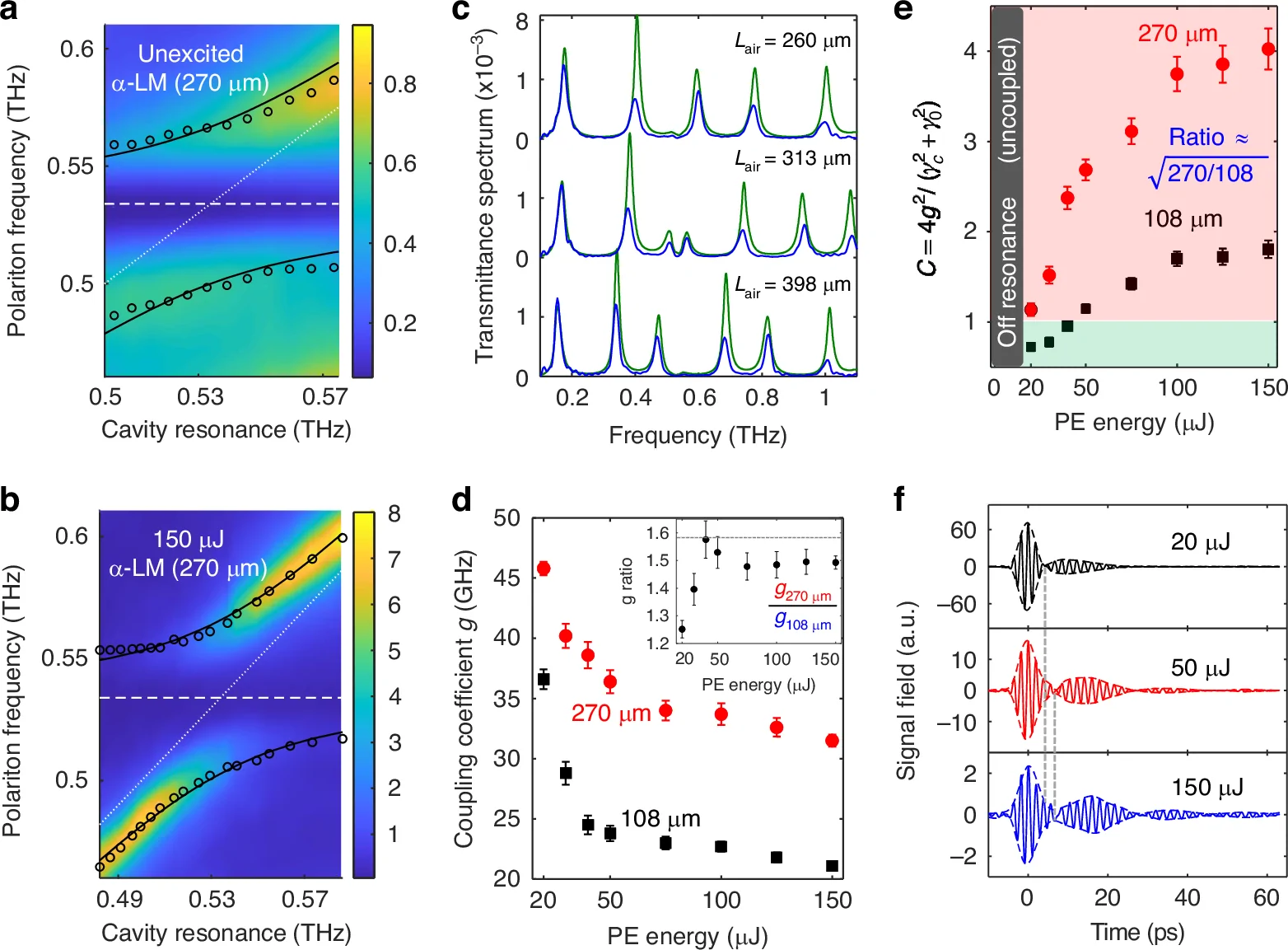
Quantifying the coupling strength of α-lactose in PE cavity. Polariton branch splitting vs. cavity resonance (νc) for (**a**) unexcited cavity, (**b**) PE cavity (150 μJ). The open circles mark the polaritons' peak frequencies. Black solid lines depict the fitted coupled oscillator model of Eq.3. Dotted and dashed lines mark the cavity resonance and *α*-LM transition respectively. **c** Exemplary transmission spectra of the PE cavity at three different detunings (blue – experimental data, green – TMM calculation). **d** Coupling coefficient(g) at various PE energies for two *α*-LM pellet thicknesses (270 μm, 108 μm). The inset shows the ratio ${g}_{270}/{g}_{108}$ of the two pellets. **e** Cooperative parameter $C=4{g}^{2}/({\gamma }_{c}^{2}+{\gamma }_{0}^{2})$ of the two pellets as a function of PE energy. **f** Time domain Rabi oscillations obtained by Fourier transformation of the spectrally filtered polaritons

While strong coupling is often quantified by the polariton splitting energy^52,65^, a more refined criterion is given by the (dimensionless) cooperative parameter $C=\frac{4{g}^{2}}{{\gamma }_{c}^{2}+{\gamma }_{0}^{2}}$^14,66^, where g is the light-matter coupling coefficient, ${\gamma }_{c}$ and ${\gamma }_{0}$ are the linewidths of the cavity mode and transition line respectively. We extract g by fitting the experimental detuning curves (in Fig. 5a, b) to the coupled oscillator model^52,66,67^:

3 $${\nu }_{{UP},{LP}}=\frac{1}{2}[{\nu }_{c}+{\nu }_{0}+i\left({\gamma }_{c}+{\gamma }_{0}\right)]\pm \sqrt{{g}^{2}+\frac{1}{4}{\left[{\nu }_{c}-{\nu }_{0}+\frac{i}{2}\left({\gamma }_{c}-{\gamma }_{0}\right)\right]}^{2}}$$

where ${{\rm{\nu }}}_{{\rm{c}}}$ is the cavity mode frequency. The parameters of the $270\,{\rm{\mu }}{\rm{m}}$ pellet of *α*-LM (${\nu }_{0}=0.53\,{\rm{THz}}$ and ${\gamma }_{0}=21\,{\rm{GHz}}$) were obtained by fitting the permittivity data derived from THz spectroscopy measurements using the Drude-Lorentz model (see SI section S5). Cavity detuning measurements were performed by translating the cavity mirror (varying only ${L}_{{air}}$) using a computer-controlled translation stage. In Figs. 5a, b we plot the polariton frequencies vs. the m=3 cavity frequency, for the unexcited cavity and PE cavity ($150\,{\rm{\mu }}{\rm{J}}$) respectively. The m=3 mode frequency (horizontal axis of Fig. 5a, b) was obtained by fitting the transmission spectra with TMM calculated spectra for different cavity lengths as exemplified by the blue (experimental) and green (TMM) curves in Fig. 5c. Using the extracted ${L}_{{air}}$ values (marked in Fig. 5c) and the background refractive index, ${\widetilde{n}}_{\alpha {LM}}=1.8$ (while discarding modulations in $\widetilde{n}$ around the $0.53\,{\rm{THz}}$ absorption line), we simulated the m=3 frequency to obtain ${\nu }_{c}$ (noted as ‘cavity resonance’ in Fig. 5a, b).

By fitting the dispersion curves of Fig. 5a, b (with ${\nu }_{c}$, ${\nu }_{0}$, ${\gamma }_{0}$ noted above and ${\gamma }_{c}$ of the empty cavity shown in Fig. 3f) to the coupled oscillators model (Eq. 3), we extract the light-matter coupling coefficient (g). Figure 5d depicts the extracted g values vs. PE energy for two different pellets:$\,270\,{\rm{\mu }}{\rm{m}}$ (red circles) and $108\,{\rm{\mu }}{\rm{m}}$ (black squares). Each data point in Fig. 5d was extracted from a designated detuning curve similar to those of Fig. 5a, b.

For the $270\,{\rm{\mu }}{\rm{m}}$ pellet, the coupling coefficient g decreases from $46\,{\rm{GHz}}$ to $34\,{\rm{GHz}}$ as the PE energy is increased from $20\,{\rm{\mu J}}$ to $70\,{\rm{\mu J}}$, followed by a more moderate decrease from $34\,{\rm{GHz}}$ to $31\,{\rm{GHz}}$ as the PE energy is further increased to $150\,{\rm{\mu J}}$. A similar trend is observed for the $108\,{\rm{\mu }}{\rm{m}}$ pellet. Analysis of the topology of the transmission reported in ref.[^20^] shows a similar trend to that of Fig. 5d (see SI section S10). Since the coupling coefficient is given by $g=\mu \sqrt{\frac{\hslash {\omega }_{0}N}{2\varepsilon {\varepsilon }_{0}V}}$, we expect the ratio $\frac{{g}_{270\mu m}}{{g}_{108\mu m}}$ of the two pellets to be $\sim \sqrt{\frac{270}{108}}=1.58$, in reasonable agreement with the experimental ratio $\left( \sim 1.5\right)$ shown in the inset of Fig. 5d. Note that this ratio is consistent with strong light-matter coupling, achieved here for PE energies $\ge 50\,{\rm{\mu }}{\rm{J}}$ energy. The strong-coupling criterion, C>1, is satisfied for the two pellets (Fig. 5e) at PE energies above $20\,{\rm{\mu }}{\rm{J}}$ for the $270\,{\rm{\mu }}{\rm{m}}$ pellet and above $50\,{\rm{\mu }}{\rm{J}}$ for the $108\,{\rm{\mu }}{\rm{m}}$ pellet. Most importantly, for PE energies below $\sim 20\,{\rm{\mu }}{\rm{J}}$ (or in the absence of PE), the system remains completely uncoupled, as the cavity frequency is far detuned from the *α*-LM transition (marked in Fig. 5e). At a PE energy of $150\,{\rm{\mu J}}$, the strong coupling parameter C (Fig. 5e) is comparable to values previously reported for a conventional mirror cavity^52^. Furthermore, the extracted parameters (*g, γ*_*c*_*, γ*_*0*_) satisfy additional criteria for strong coupling reported in the literature such as: ${\varOmega }_{R} > {\gamma }_{c},{\gamma }_{0}$^68^ and ${\varOmega }_{R} > \frac{{\gamma }_{c}+{\gamma }_{0}}{2}$^69^, providing further evidence that the system has transitioned into the strong-coupling regime upon PE. We note that the ratio $\frac{{g}_{270\mu m}}{{g}_{108\mu m}}\approx 1.5$ is slightly lower than the theoretically expected value of 1.58. This deviation is attributed to the fixed, non-tunable positioning of the pellets on the Si cavity mirror, which reduces their spatial overlap with the m=3 cavity mode profile^70^.

The above-noted decrease in g indicates a slowing down of the energy exchange in the system (Rabi oscillations), as can be directly observed in the time domain signal, shown in Fig. 5f. Here, the frequency-domain signal was spectrally filtered to isolate the polaritons in the range $[0.44\,{\rm{THz}},\,0.64\,{\rm{THz}}]$, followed by an inverse Fourier transformation back into the time domain^52^. As the PE energy is increased $(20\,{\rm{\mu }}{\rm{J}}\to 50\,{\rm{\mu }}{\rm{J}}\to 150\,{\rm{\mu }}{\rm{J}})$, the Rabi frequency decreases $(84\,{\rm{GHz}}\to 69\,{\rm{GHz}}\to 59\,{\rm{GHz}})$ and the polaritons’ linewidths become narrower. This manifest as extended beat period (dashed gray lines) and extended lifetime of the Rabi oscillations. Put together, the coupling strength of the system increases from $C=1.14\to 2.68\to 4.02$ with the increasing PE energy as shown in Fig. 5e.

## Conclusions and outlook

We have presented an optically switchable Fabry-Pérot cavity that enables ‘on-demand’ ultrafast triggering of strong light–matter coupling. A material placed within the cavity experiences an abrupt change in the properties of the photonic environment it is embedded within: from a low-Q, off-resonance conditions (“bad cavity”, uncoupled state) to the high-Q, on-resonant regime (“good cavity”, strong light-matter-coupling). This optically triggered transition occurs on a time scale of $\sim 4\,{\rm{ps}}$, and persists for a few ns duration, governed by the recombination time of the Si. Importantly, the switching from the uncoupled to strong-coupling occurs on timescales shorter than the cavity photon lifetime ($\sim 45\,{\rm{ps}}$ in this work) and in our case also shorter than the characteristic time for coherent energy exchange between the photonic and molecular system (Rabi period, ${1/\varOmega }_{R} \sim 17\,{\rm{ps}}$). While the excitation and decay dynamics of polariton populations have been widely studied, our scheme aims for accessing the dynamics of the polaritonic state themselves, e.g. the temporal onset of polariton formation, yet to be explored.

Most importantly, the presented cavity and control scheme are designed to fulfil a key requirement for polaritonic chemistry endeavors: the switching mechanism exclusively modifies the photonic environment, leaving the embedded molecular or material system unperturbed. Currently, the lower limit of the “cavity ON” duration is governed by the few ns recombination time of Si. This time-scale is suited for strong-coupling-induced perturbation of relaxation and energy redistribution dynamics of librational modes, intermolecular vibrations and collective solvent modes that govern, for example, charge transfer recombination.

Extending the duration in which strong-coupling conditions persist, beyond the few ns time is possible by, e.g. using a series of short PE pulses or a long duration pulse.

We note that our ongoing efforts aim to enable on-demand switching with a controlled duration over which strong coupling occurs, potentially reducing the temporal window to $\sim 10{\rm{ps}}$. Such temporal control could provide access to the microscopic time scales of reaction kinetics under strong-coupling conditions.

We emphasize that while our results demonstrate the capability to induce strong light–matter coupling on ultrashort time scales, the present linear THz spectroscopic data alone do not establish modifications of the intrinsic chemical or physical potential energy surfaces of the cavity-embedded α-LM.

## Methods

### Silicon cavity configuration

We have used $12\,\mu {\rm{m}}$ thick high-resistivity, double-sided polished Si wafers purchased from Virginia Semiconductors as cavity mirrors. The Si wafers were mounted on a homemade aluminium holder such that their center is free-standing. One of the Si holder was mounted on a computer-controlled translation state to enable cavity detuning. The cavity plates were made parallel by monitoring the reflection of a CW He-Ne laser. To prepare the *α*-LM pellets we pressed *α*-LM crystalline powder (as purchased from Sigma-Aldrich) using a pressing die (diameter $20\,{\rm{mm}}$) at a pressure of $220\,{\rm{kN}}$ for 20 min. For $108\,\mu {\rm{m}}$ and $270\,\mu {\rm{m}}$ thick pellets we weighted $48\,{\rm{mg}}$ and $120\,{\rm{mg}}$ of *α*-LM powder respectively. The pellets were mounted on the Si mirror using minimal amount of commercially available fast glue, at the edges of the pellets.

### Transfer matrix calculations

To simulate the optical response of the multilayer structure, we employed the transfer matrix method (TMM), which enables the calculation of frequency dependent transmission by systematically combining the optical responses of individual layers^71^.

The system under study consists of six layers: incident air medium (medium 1), Si-mirror (medium 2), air-filled cavity layer (medium 3), *α*-LM pellet (medium 4), Si-mirror (medium 5) and air at the output of the cavity (medium 6). At each frequency$\,\nu$, the free space wave vector is defined as ${k}_{0}=2\pi \nu /c$, where c is the speed of light. In the simulation, we used the complex refractive index $\widetilde{n}\left(\omega \right)$ of Si (unexcited and PE with varying pulse energies) shown in Fig. 2b and the complex refractive index of the *α*-LM pellet, shown in Fig. 4b.

For each interface between the adjacent media a and b, the Fresnel reflection and transmission coefficients are given by ${\widetilde{r}}_{{ab}}=\frac{{\tilde{n}}_{a}-{\tilde{n}}_{b}}{{\tilde{n}}_{a}+{\tilde{n}}_{b}}$ and ${\widetilde{t}}_{{ab}}=\frac{2{\tilde{n}}_{a}}{{\tilde{n}}_{a}+{\tilde{n}}_{b}}$

The propagation matrix ${P}_{a}$ (the phase accumulated through the layer) for layer a of thickness ${d}_{a}$ and complex refractive index ${\widetilde{n}}_{a}$ is expressed as

$${P}_{a}=\left[\begin{array}{cc}{e}^{-i{k}_{0}{\tilde{n}}_{a}{d}_{a}} & 0\\ 0 & {e}^{i{k}_{0}{\tilde{n}}_{a}{d}_{a}}\end{array}\right]$$

The interface matrix (accounting for transmission and reflection) between layers *a* and *b* is:

$${T}_{{ab}}=\frac{1}{{\widetilde{t}}_{{ab}}}\left[\begin{array}{cc}1 & {\widetilde{r}}_{{ab}}\\ {\widetilde{r}}_{{ab}} & 1\end{array}\right]$$

The total transfer matrix M for the complete layer architecture is calculated as the product of the interface and propagation matrices:

$$M={T}_{12}{.P}_{2}{.T}_{23}{.P}_{3}{.T}_{34}.{P}_{3}.{T}_{45}{.P}_{4}{.T}_{56}$$

The complex transmission ($\widetilde{{\rm{t}}}$) coefficient is the reciprocal value of the ${{\rm{M}}}_{11}$ matrix element: $\widetilde{{\rm{t}}}=\frac{1}{{{\rm{M}}}_{11}}$

The power transmittance (${\rm{T}}$) is given by ${\rm{T}}={\left|\widetilde{{\rm{t}}}\right|}^{2}$

The TMM calculations are performed for each frequency selectively.

## Supplementary information

Supplementary Information

## Data Availability

All of the data supporting the findings of this study are available within the article and Supplementary information file (SI). All raw data and analysis scripts are available upon request from the authors.
