# Supplementary material for: Ultrafast triggering of strong coupling in a semiconductor terahertz Fabry-Pérot cavity
Source: Light Sci Appl. 2026 Aug 19;15:351. doi: 10.1038/s41377-026-02379-2 (PMC13490365; doi:10.1038/s41377-026-02379-2)
Supplement: Supplementary file 1 [file 41377_2026_2379_MOESM1_ESM.pdf]

Supplementary Information

Ultrafast Triggering of Strong Coupling in a Semiconductor Terahertz Fabry-Pérot Cavity

Soumitra Hazra[1,2], Hanna Turchinsky[1,2], Tal Schwartz[1,2], and Sharly Fleischer[1,2]

[1]Raymond and Beverly Sackler Faculty of Exact Sciences, School of Chemistry, Tel Aviv University, Tel Aviv 6997801, Israel
[2]Tel Aviv University Center for Light-Matter Interaction, Tel Aviv 6997801, Israel
Email: soumitra.hazra89@gmail.com, sharlyf@tauex.tau.ac.il

Section S1: Calculation of THz complex refractive index of the silicon wafer

For extracting the complex refractive index of the Si wafer in the THz frequency range, we have measured the THz transmission through the silicon wafer ($\tilde{E}_{sample}$) and through air ($\tilde{E}_{reference}$) as reference. The ratio of the Fourier transform of the THz field provides the experimental complex transmission i.e. $\tilde{T}_{exp}(\omega) = \frac{\tilde{E}_{sample}(\omega)}{\tilde{E}_{reference}(\omega)}$. The theoretical transfer function of the Si wafer includes the Fresnel reflection and transmission coefficient of the Si-air interface given by $\tilde{r}_{ij} = \frac{\tilde{n}_i - \tilde{n}_j}{\tilde{n}_i + \tilde{n}_j}$, $\tilde{t}_{ij} = \frac{2\tilde{n}_i}{\tilde{n}_i + \tilde{n}_j}$, where $\tilde{n}_i$ and $\tilde{n}_j$ are the complex refractive index of the respective media. Additionally, the propagation of the field through the Si wafer is described by $\tilde{P}_j = e^{-i\tilde{n}_j d_j k_0}$, where d_j is the thickness of the Si wafer and k_0 is the free space wavevector. The Fabry-Pérot reflection term inside the Si wafer is given by, $\widetilde{FP}_{ijk} = \frac{1}{1 - \tilde{r}_{ji}\tilde{r}_{jk}\tilde{P}^2{}_j}$. These components, as illustrated in Fig. S1a, altogether define the theoretical transfer function ($\tilde{T}_{theo}$) with air as the reference, which is presented in eq. 1 in the main text.

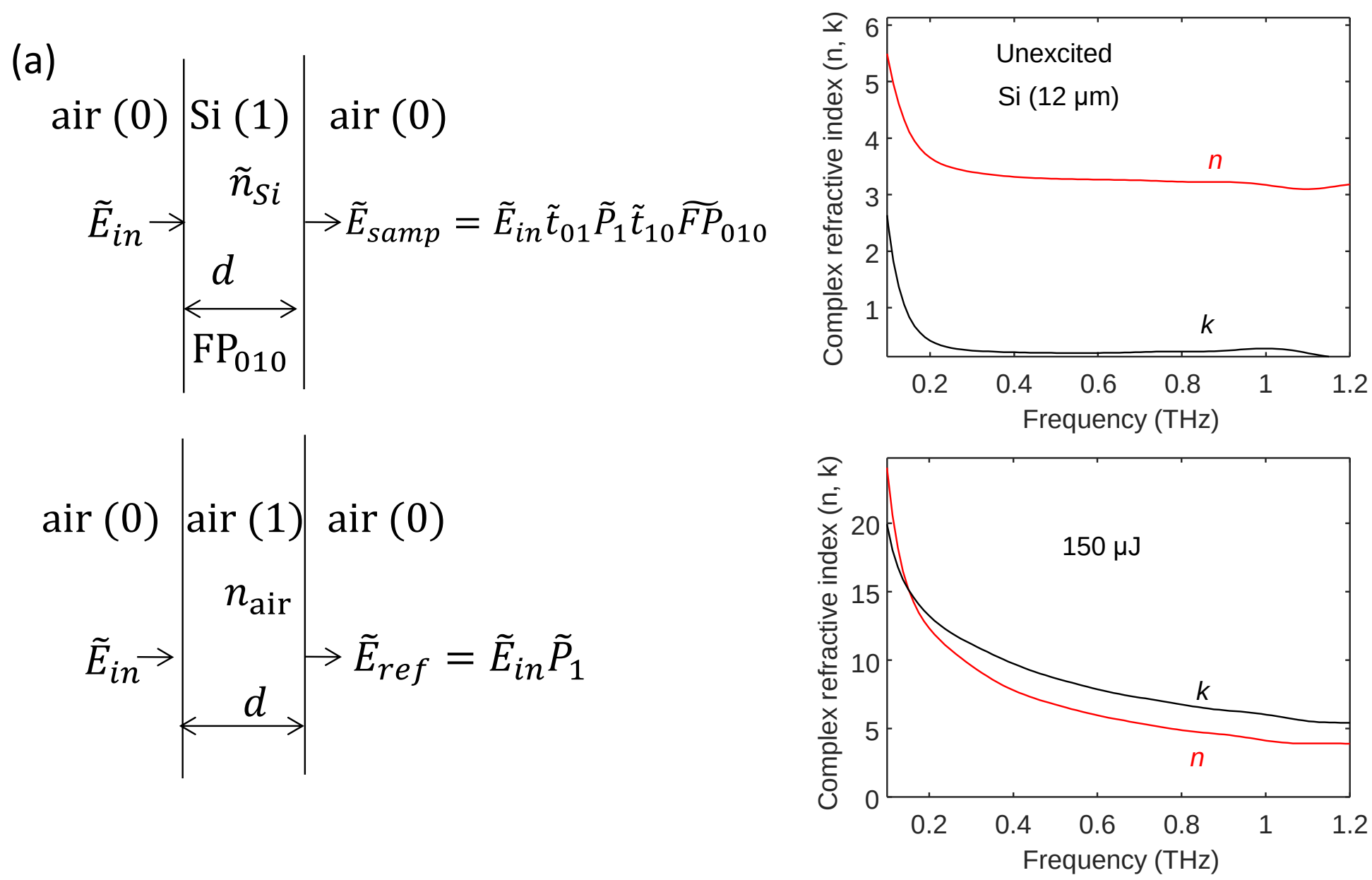

Figure S1: (a) Sample and reference settings for building the theoretical transfer function for the Si wafer [1]. (b) and (c) show the extracted refractive index coefficients (real part $[n]$ in red, imaginary part $[k]$ in black) of unexcited and PE (with 150 μJ energy) Si wafer respectively.

The thickness of the thin silicon wafer was mechanically measured as $d = 12$ μm, using a micrometer screw gauge. Numerical minimization of the difference between the experimental and theoretical transfer function ($\tilde{T}_{exp} - \tilde{T}_{theo}$) yields the complex refractive index $\tilde{n} = n_{Si} + ik_{Si}$. For the optimization, we have used total variation technique based on the Nelder-Mead algorithm [2]. The extracted frequency-dependent refractive indices using our home-built code were found identical to those extracted using Nelly, an open-source package for extracting the complex THz refractive index [3].

Section S2: Reflectivity of Si wafer

The power reflectivity (R) of the ($d = 12$ μm) Si wafer was calculated as [4]:

$$R = \left|\frac{\tilde{r}_{10}+\tilde{r}_{01}e^{-i\phi}}{1+\tilde{r}_{10}\tilde{r}_{01}e^{-i\phi}}\right|^2 \qquad \text{(s1)}$$

We note the importance of accounting for multiple internal reflections and interference effects within the thin, subwavelength Si wafer.

Here, $\tilde{r}_{10}$ and $\tilde{r}_{01}$ are the complex Fresnel reflection coefficient at the air-Si and Si-air interfaces respectively. The phase delay due to round-trip propagation within the Si layer is given by $\phi = 4\pi\tilde{n}_{Si}d/\lambda$, where $\tilde{n}_{Si}$ is the complex refractive index of Si and λ is the THz wavelength in vacuum (air). The calculated power reflectivity of the unexcited and PE Si at various PE energies is shown in Fig. 2c.

Section S3: Phase of reflection at the interface of the cavity

We calculated the frequency-dependent complex reflection coefficient ($\tilde{r}_{ij} = \frac{\tilde{n}_i-\tilde{n}_j}{\tilde{n}_i+\tilde{n}_j}$) at the air-Si ($I_2, I_3$) and Si-air ($I_1, I_4$) interfaces using the complex refractive index of the Si wafer, as shown in Fig.2b. The phase of the reflection coefficient defined as $\phi_{ij} = \arg(\tilde{r}_{ij})$ is approximately $\phi_{air-Si} \approx \pi$ and $\phi_{Si-air} \approx 0$ for both unexcited and PE Si, as illustrated in Fig. S3.

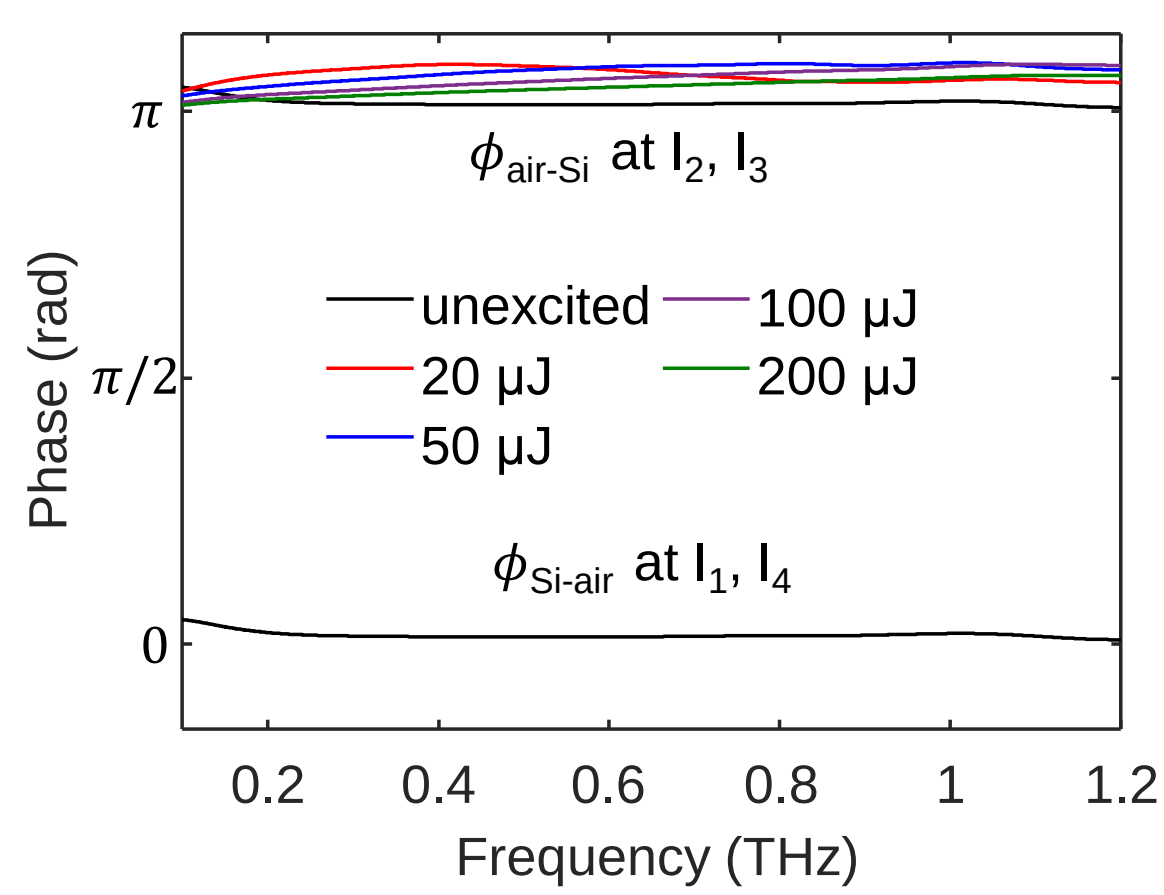

Figure S3: The reflection at the air-Si (ϕ_{air-Si} at I_2, I_3) and Si-air (ϕ_{Si-air} at I_1, I_4) interfaces for PE energies spanning the range of photoexcitation energies used in this work.

Section S4: PE-Induced Redshift at small cavity length

As the main reason for the redshift is attributed to the phase-shift at the air-Si interface, the effect of the latter on the detuning magnitude is larger at small cavity length. This is exemplified here for a switchable Si cavity with $L_{air} = 240$ μm (~4 times smaller than the cavity length used in Fig. 3 of the main text). Here the unexcited m=1 cavity frequency is 750 GHz and upon PE a large redshift is observed, down to 590 GHz.

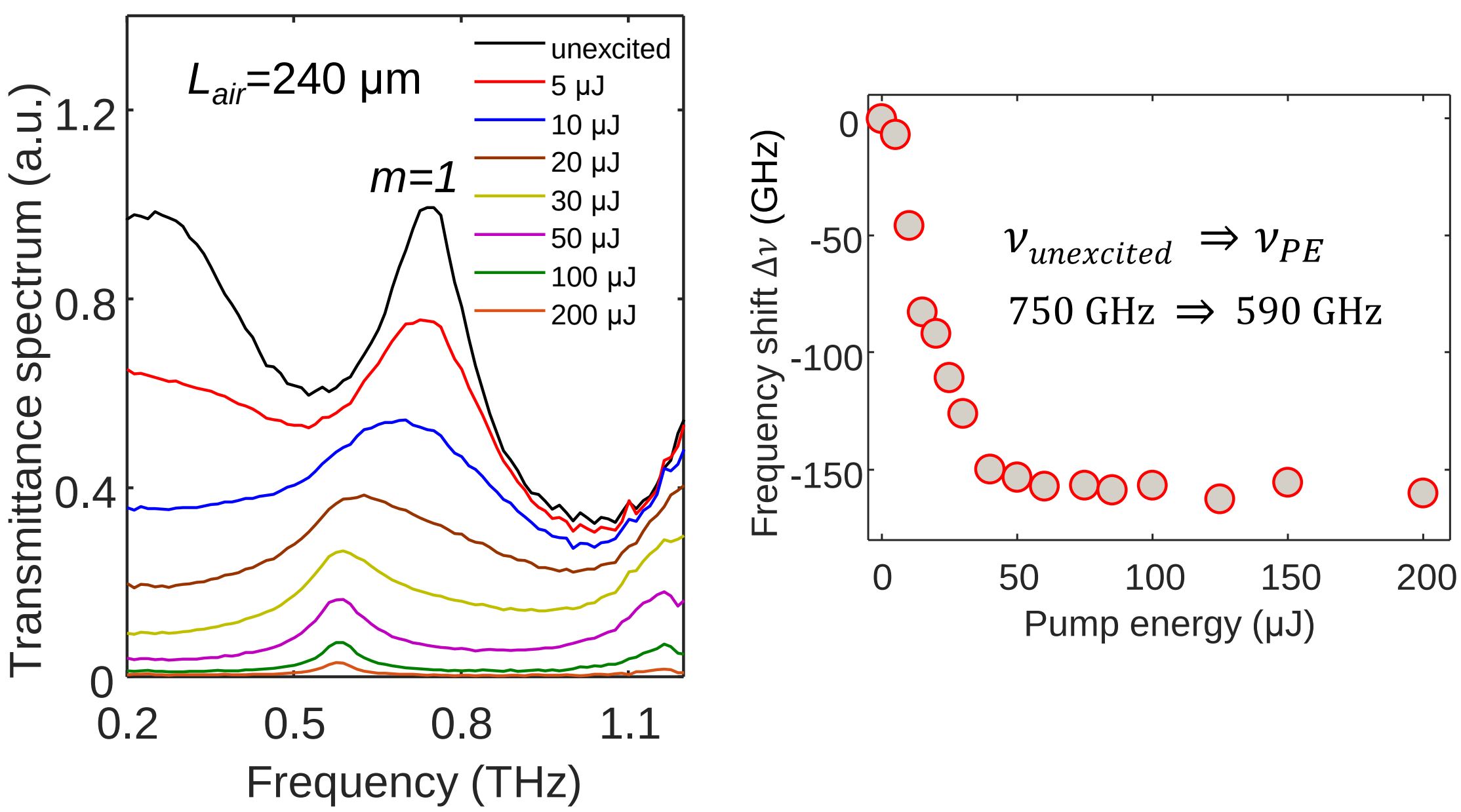

Figure S4: (a) The transmission of the empty cavity for cavity length $L_{air} = 240$ μm where, the $m = 1$ (unexcited) cavity frequency is 750 GHz. The cavity modes are redshifted with increasing the PE energy. (b) Frequency redshift of the $m = 1$ cavity mode at varying PE energies.

Section S5: Complex permittivity of α-Lactose crystal

Using the complex refractive index ($\tilde{n} = n + ik$) as shown in Fig.4b, the complex permittivity was extracted using the relations: $\epsilon_1 = n^2 - k^2$ and $\epsilon_2 = 2nk$. The complex permittivity was fitted with the Drude-Lorentz model with one vibrational transition [5]:

$$\epsilon_{\alpha L}(\omega) = \epsilon_\infty + \frac{\omega_p^2}{{\omega_0}^2 - \omega^2 - i\gamma\omega} \quad \text{(s2)}$$

where $\epsilon_\infty = 3.2$ is the background dielectric constant, $\omega_0 = 0.53\ \text{THz}$ is the vibrational mode, $\gamma = 21\ \text{GHz}$ is the linewidth and $\omega_p = 110\ \text{GHz}$ is the plasma frequency. The complex permittivity of the α-lactose with the Drude-Lorentz fit is shown in Fig. S5.

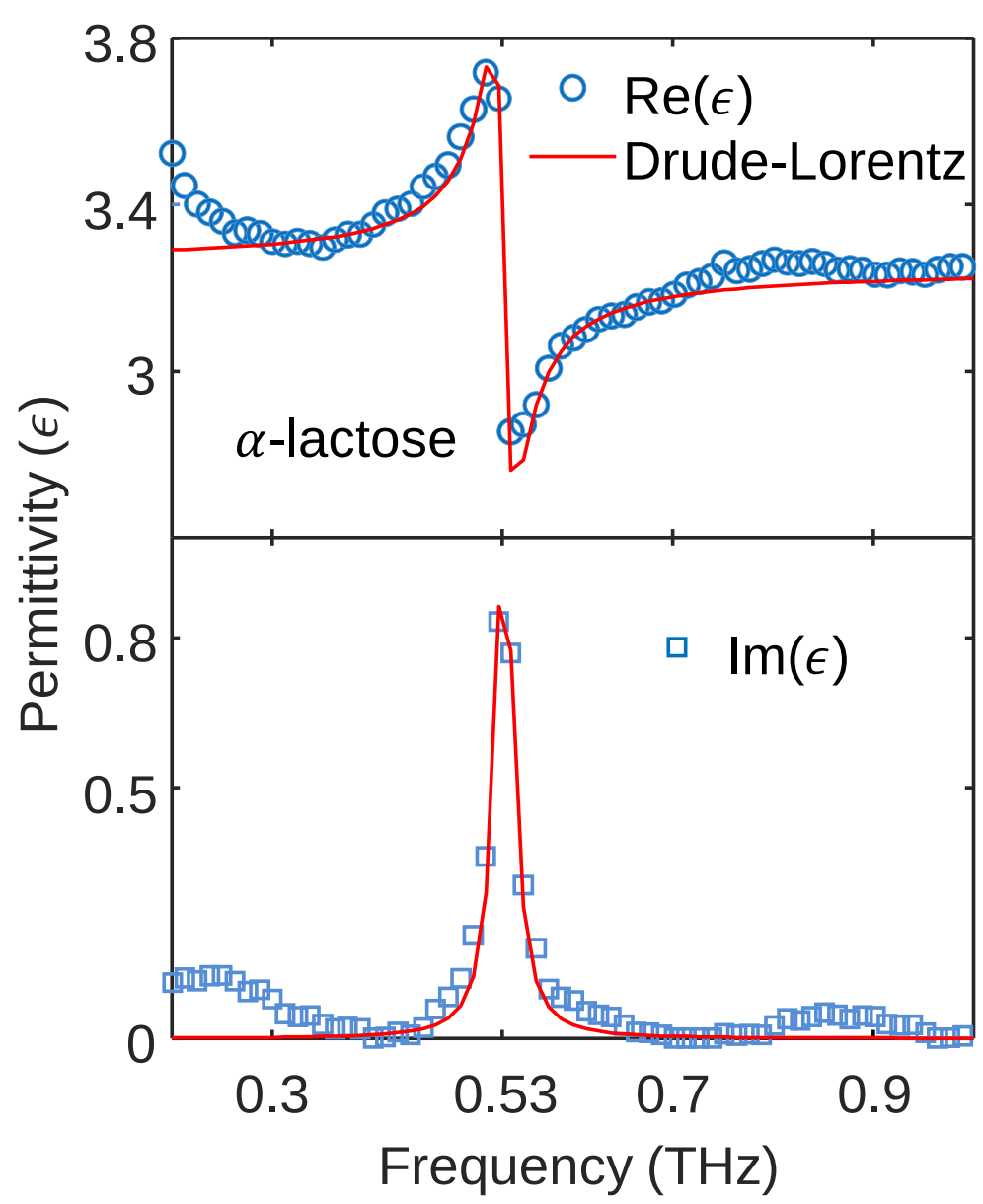

Figure S5: The complex permittivity of the α-lactose pellet of thickness $270\ \mu m$.
The solid line is the Drude-Lorentz model fit (eq. s2).

Section S6: Thermal effect of photoexcitation on cavity transmission

Since long-time irradiation of the Si wafers by the 800 nm PE pulse train at the repetition rate of our laser ($1\ \mathrm{KHz}$) may lead to accumulation of heat and elevated temperature, we compared the transmission of the unexcited cavity to that of an excited cavity. Here the PE pulse arrives at a delay of $60\ \mathrm{ps}$ past the THz probe ($\sim 1\ \mathrm{ms}$ past recombination), yet affected by the (possibly) accumulated temperatures. As readily observed, the unexcited and the $150\ \mu\mathrm{J}$ PE transmission spectra are in very good agreement, reassuring that thermally-induced changes in the optical parameters of the Si are negligibly small.

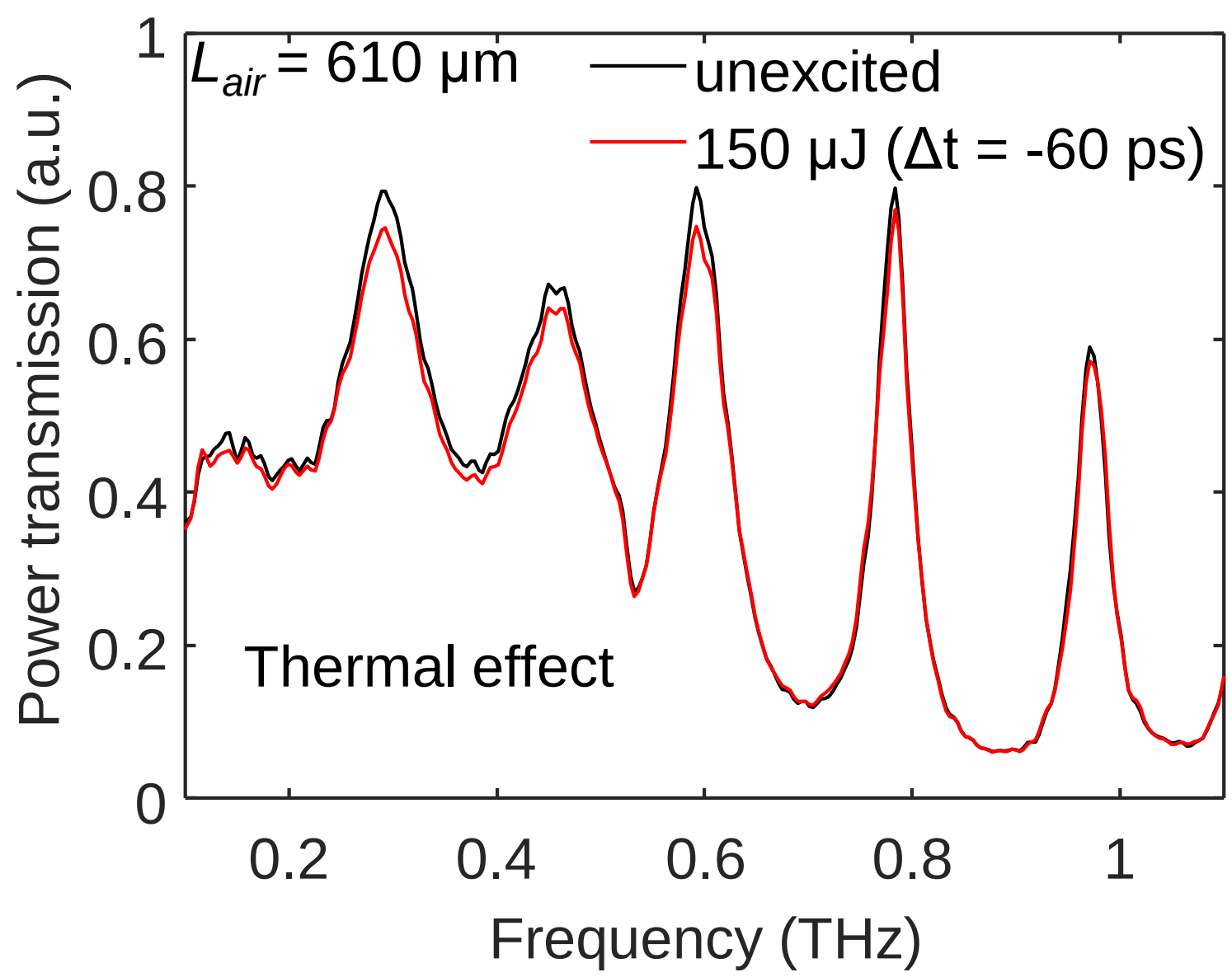

Figure S6: The power transmission spectrum of the Si FP cavity with $108\ \mu m$ α-LM pellet inside for unexcited and PE at pump energy $150\ \mu\mathrm{J}$, pump delay $\Delta t = -60\ \mathrm{ps}$.

Section S7: Permittivity of photoexcited Si

In order to assess the metallicity of the photoexcited Si mirrors we provide in Fig. S7 the permittivity values of our ultrathin Si wafers following photoexcitation by several pulse energies. As the photoexcitation energy (fluence) is increased, the real part decrease and the imaginary part increase, as the Si layer transforms to the metallic state.

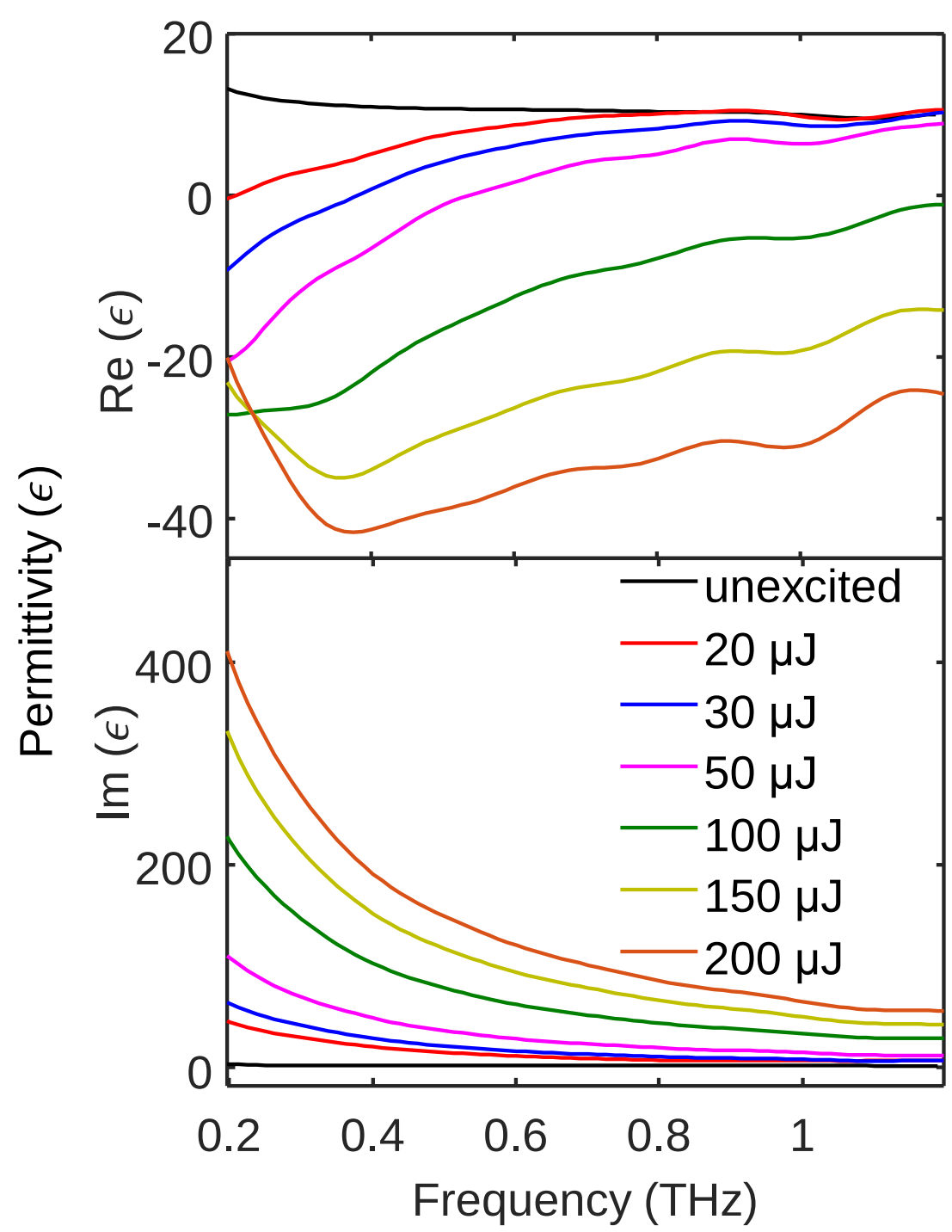

Figure S7: The complex permittivity of unexcited and photoexcited Si with different pump energies.

Section S8: Phase considerations for Fabry-Pérot resonances (why $\boldsymbol{m \equiv q - 1}$)

The resonance frequency of a Fabry-Pérot cavity is determined by the total round-trip phase accumulated by the field, ϕ_{rt}, which must satisfy the condition $\phi_{rt} = 2\pi q$ (where q is an integer). This phase consists of the propagation phase (ϕ_{prop} associated with physical length) and the reflection phases ($\phi_{r1} + \phi_{r2}$) at the cavity mirrors:

$\phi_{rt} = \phi_{prop} + \phi_{r1} + \phi_{r2} = 2\pi q$ (eq.2 in main text file)

In a FP cavity composed of two metallic mirrors at a distance L apart, the reflection phases at the mirrors are $\phi_{r1} = \phi_{r2} = \pi$ and the propagation phase is $\phi_{prop} = \frac{4\pi L}{\lambda}$.

Plugged into (eq.2) this gives: $\phi_{rt} = \frac{4\pi L}{\lambda} + \pi + \pi = 2\pi q \quad \rightarrow \quad \frac{4\pi L}{\lambda} = 2\pi(q - 1)$

Denoting $m \equiv q - 1$, we arrive at the well-known expression of the FP resonances with $\lambda_m = \frac{2L}{m}$ and frequency-wise: $\nu_m = \frac{c}{2L} m$, where m is the mode number and c- the speed of light.

Note that the above formulation aligns with the enhanced transmission observed in zero-order ($m = 0$) Fabry-Pérot cavity [6], where the distance between the mirrors is significantly shorter than the wavelength ($L \approx 0$). In this case, the propagation phase is $\phi_{prop} \approx 0$ and the roundtrip phase condition of eq.2 is:

$$\phi_{rt} = \underbrace{\phi_{prop}}_{0} + \phi_{r1} + \phi_{r2} = 2\pi(\underbrace{m}_{0} + 1)$$

correctly accounting for the reflection phases $\phi_{r1} = \phi_{r2} = \pi$

Section S9: Comparison of selective transfer function calcualtions with TMM and experimental data

In order to verify that configuration (iv) noted in the main paper (I_1/I_3 and I_2/I_4) indeed dominates the THz transmission spectrum of the cavity, we performed analytical transfer function calculations for all of the possible configurations (i-iv) and compared them with the TMM simulation results and with the experimental data. The results are summarized in Fig. S8.

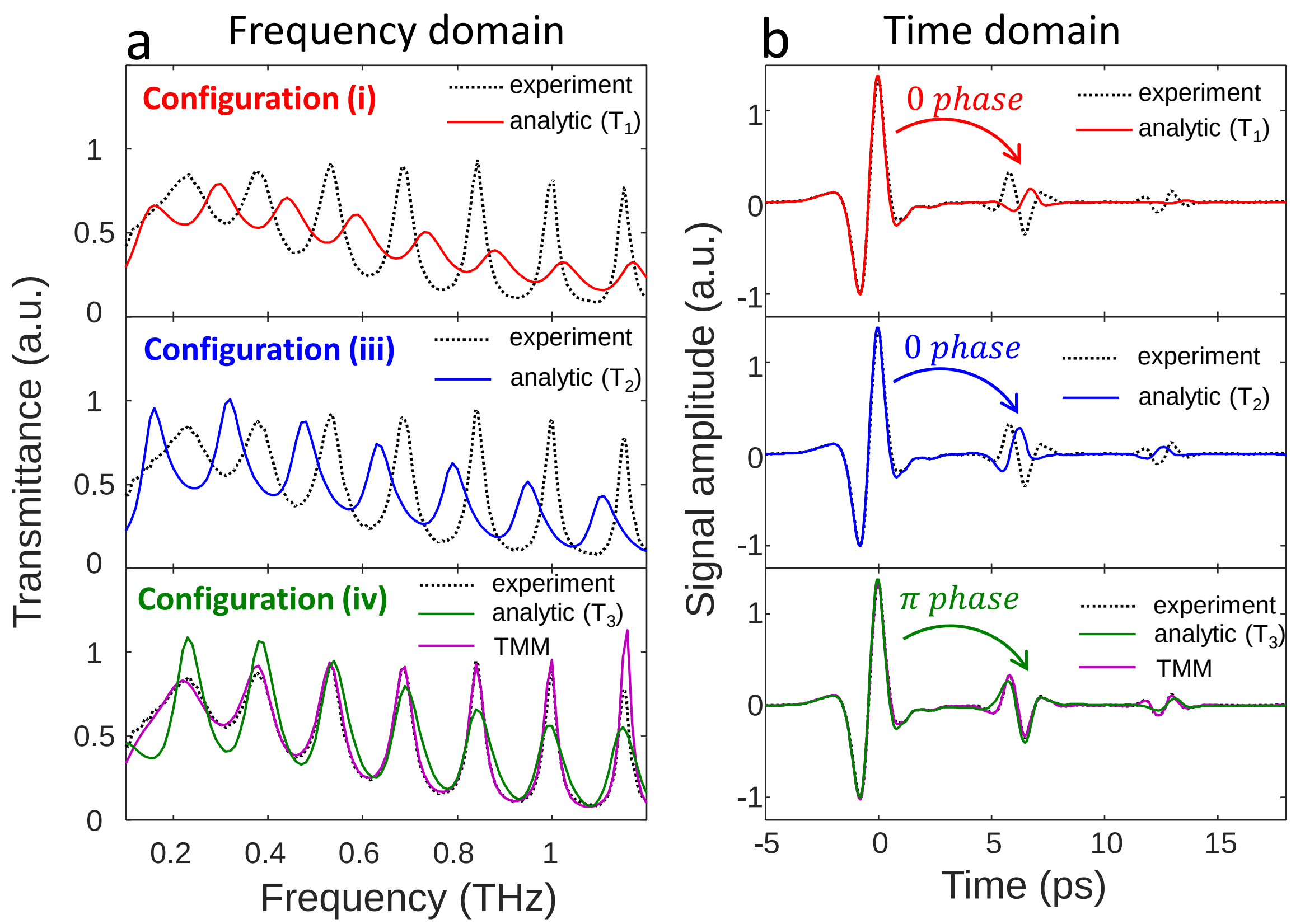

Figure S8: Analytical Transfer Function calculations for selective cavity configurations (color-coded) accommodated by the Si-air-Si stack with $L_{air} = 925\ \mu\text{m}$. (a) Frequency domain transmission spectra. (b) Time domain THz transmission obtained by Fourier transformation of the product of the (complex) transfer functions shown in (a) with the incident THz field. The TMM calculation of the stack are colored pink, with excellent agreement the transfer function results for configuration (iv). The experimental signal is depicted by the dotted black lines for comparison.

Figure S8a shows the transmission spectra for cavity configurations (i, iii, iv) presented in the main paper text. As can be readily observed, the analytical transfer function for configuration (iv), shown by the green curve in the lower panel of Fig. S8a (T_3) is in excellent agreement with

both the TMM (pink) and experimental data (dotted black). Note that configurations (i, iii) are practically not represented in the experimental and TMM spectra.

The time domain spectra of Fig.S8b further validates that configuration (iv) is the dominant and essentially the only configuration that is represented in the experiment and TMM, as can be seen by the π phase shift between consecutive reflection transients (marked in the figure). In contrast, configurations (i, iii) show phase-similar transients (marked 0 phase in the figure).

<u>Calculation details</u>

The analytical transfer function of the empty Si Fabry–Pérot (FP) cavity, includes a combination of Fresnel coefficients and propagation factors accumulated by the THz field through its multiple roundtrip oscillation. Considering the reflection from an interface between two media a and b designated by complex refractive indices $\tilde{n}_a$ and $\tilde{n}_b$, the Fresnel reflection and transmission coefficients at the interface is given by [7]:

$\tilde{r}_{ab} = (\tilde{n}_a - \tilde{n}_b)/(\tilde{n}_a + \tilde{n}_b)$ and $\tilde{t}_{ab} = 2\tilde{n}_a/(\tilde{n}_a + \tilde{n}_b)$ respectively .

The total transmitted field of the FP cavity (Si-air-Si) is obtained by summing up all possible paths including the directly transmitted field and multiple FP reflections at the selective interfaces. The resulting transfer function can be written as

$$\tilde{T} = \tilde{t}_{air,Si} e^{i\frac{(\tilde{n}_{Si}-1)\omega L_{Si}}{c}} \tilde{t}_{Si,air} \tilde{t}_{air,Si} e^{i\frac{(\tilde{n}_{Si}-1)\omega L_{Si}}{c}} \tilde{t}_{Si,air} \widetilde{FP} \qquad \text{(s3)}$$

where, $\widetilde{FP}$ stands for the Fabry–Pérot reflections between the selective interfaces for a given cavity configuration.

For configuration (i), the transmission T_1 is obtained by substituting the corresponding $\widetilde{FP}$ factor into eq.(s3), where

$$\widetilde{FP} = 1/\left(1 - \tilde{r}_{Si,air}^2 \tilde{t}_{Si,air}^2 \tilde{t}_{air,Si}^2 e^{i\frac{2\omega(2L_{Si}\tilde{n}_{Si}+L_{air})}{c}}\right)$$

For configuration (iii), the transmission T_2, is similarly obtained using

$$\widetilde{FP} = 1/\left(1 - \tilde{r}_{air,Si}^{2} e^{i\frac{2\omega L_{air}}{c}}\right)$$

For configuration (iv), the transmission T_3 is calculated with

$$\widetilde{FP} = 1/\left(1 - \tilde{r}_{air,Si}\tilde{r}_{Si,air}\tilde{t}_{air,Si}\tilde{t}_{Si,air} e^{i\frac{2\omega(L_{Si}\tilde{n}_{Si}+L_{air})}{c}}\right)$$

Configuration (ii) corresponds to a cavity mode bound to oscillate between the inner and outer Si-air interface within each Si wafer separately. The $\widetilde{FP}$ term for this configuration is $\widetilde{FP}$=1/($1 - \tilde{r}_{Si,air}^{2} e^{i\frac{2\omega L_{Si}\tilde{n}_{Si}}{c}}$). These reflections have a roundtrip time of ~ 270 fs, with the first resonance mode appearing at ~ 3.6 THz, far beyond our usable THz bandwidth. Its contribution, however, manifests as a background amplitude associated with zeroth-order modes. Accordingly, this contribution is included as an additive background in the calculations for configurations (i), (iii), and (iv).

The transmitted time-domain signal (shown in Fig. S8b) is obtained by applying an inverse Fourier transform to the product of the complex cavity transfer function $\tilde{T}(\omega)$ and the incident complex THz electric field $\tilde{E}_{in}(\omega)$,

$$E_t(t) = IFFT\ [\tilde{T}(\omega) \cdot \tilde{E}_{in}(\omega)]$$

Section S10: Analysis of the topology of complex transmission

In this section we provide a complementary approach utilized for analyzing the onset of strong-coupling relying on the topology of the complex transmission [8–10]. The topology of the complex transmission for the α-LM (270 μm thick pellet) embedded within the PE Si cavity was analyzed over the frequency range $0.48 - 0.6$ THz for different PE energies. The analysis is performed by plotting the real part vs. the imaginary part of the complex transmission (T) as shown in Fig. S9b.

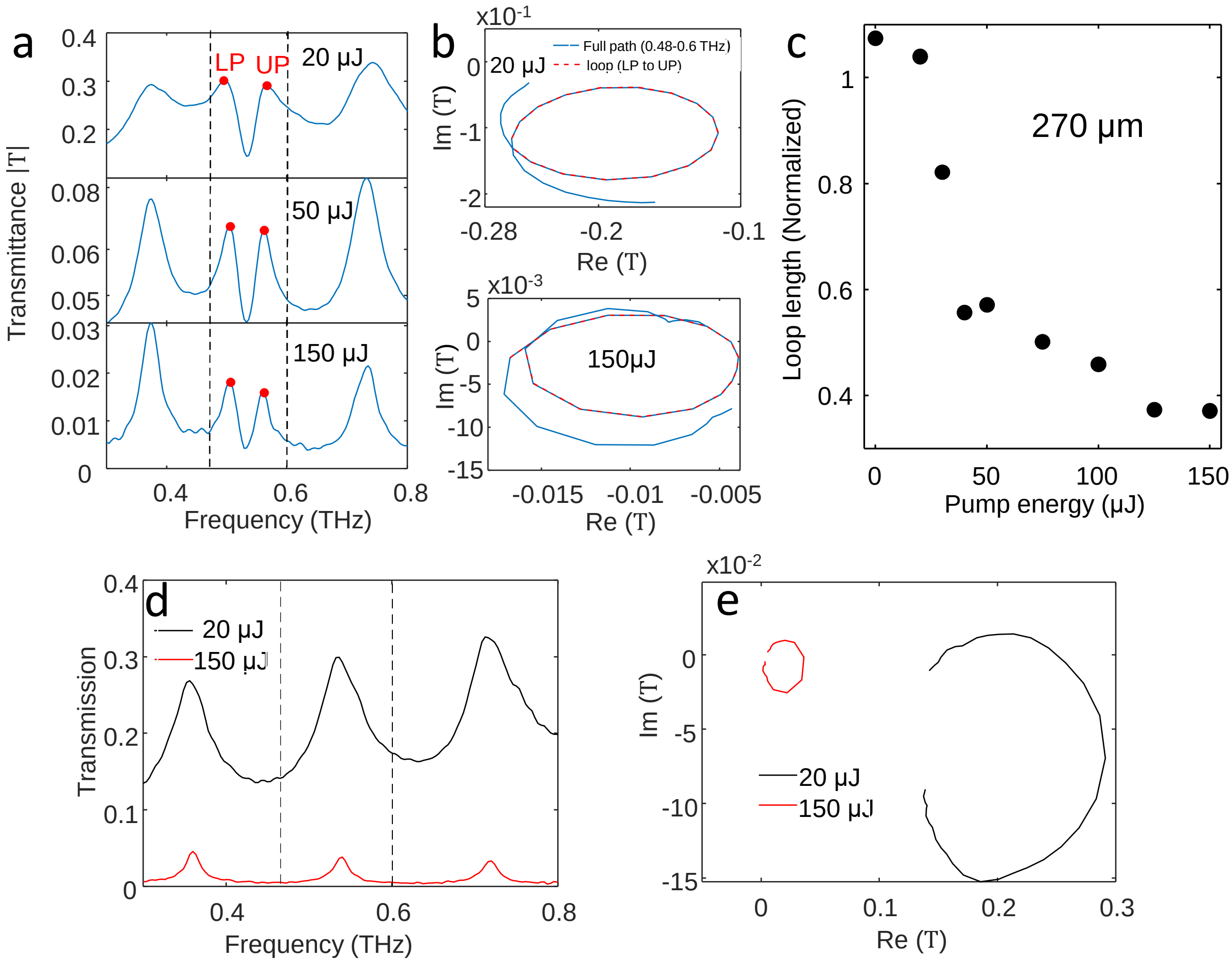

Figure S9: (a) Transmission spectra of the photoexcited (PE) cavity at zero detuning for pump energies of 20 μJ, 50 μJ and 150 μJ. The red points mark the positions of the lower (LP) and upper (UP) polariton branches. (b) Topology of the complex transmission for PE energies of 20 μJ and 150 μJ. The solid blue curves correspond to the complex path in the 0.48–0.60 THz frequency range, while the red dashed curves highlight the frequency interval between the LP and UP branches, identified at the intersection point. (c) Secondary loop length (red dashed loops in Fig. b) as a function of PE energy. (d) and (e) Transmission amplitude and the complex plane representation of the transmission of the empty cavity following PE with 20 μJ and 150 μJ.

The loop formed by the intersection of the blue path ($0.48 - 0.6$ THz region) with itself, and is typically referred to as "secondary loop", the length of which corresponds to the Rabi splitting energy of the polaritons. The intersection position (forming the loop) is used to identify the polariton frequencies (marked by red points in Fig. S9a). Figure S9c depicts the loop length as a function of PE energy, showing a rapid decrease between 20 μJ to 40 μJ, followed by a slower reduction for higher PE energies. This trend is consistent with the sharp decrease in coupling coefficient g depicted in Fig. 5d of the main paper file. We note that in order to implement the complex transmission analysis and to quantify the relative loop lengths for different PE energies, we have normalized the secondary loop (in between the polaritons) by the length of the loop formed by the $m = 3$ mode of the empty cavity tuned to 0.53 THz under the same PE energy. Figures S9d depicts the transmission of the empty cavity excited by 20 μJ and 150 μJ. The complex topology analysis shows the corresponding broken loops for the two PE energies (Fig. S9e), with significant difference in length, as expected from the reduced transmission at higher PE energies.

Section S11: Versatility of the switchable cavity and damage threshold limitations.

The versatility of the presented switchable cavity to accommodate a range of material systems with different transition frequencies is provided in the table below. We used the closed simplified expressions for $\nu_{\text{unexcited}}$ and ν_{excited} provided in the main article.

L_{air}	$m = 1$		$m = 2$		$m = 3$		$m = 4$	
	$\nu_{\text{un}} \rightarrow$	ν_{ex}	$\nu_{\text{un}} \rightarrow$	ν_{ex}	$\nu_{\text{un}} \rightarrow$	ν_{ex}	$\nu_{\text{un}} \rightarrow$	ν_{ex}
500μm	0.416 THz	0.3 THz	0.693 THz	0.6 THz	0.97 THz	0.9 THz	1.248 THz	1.2 THz
250μm	0.773 THz	0.6 THz	1.29 THz	1.2 THz	1.804 THz	1.8 THz	2.32 THz	2.4 THz
125μm	1.355 THz	1.2 THz	2.26 THz	2.4 THz	3.16 THz	3.6 THz	4.067 THz	4.8 THz

As can be seen, by varying the cavity parameters one can access a broad range of THz frequencies and even obtain blueshifts in frequency upon photoexcitation (colored blue).

References

[1] L. Duvillaret, F. Garet, and J.-L. Coutaz, *A Reliable Method for Extraction of Material Parameters in Terahertz Time-Domain Spectroscopy*, IEEE J. Sel. Top. Quantum Electron. **2**, 739 (1996).

[2] I. Pupeza, R. Wilk, and M. Koch, *Highly Accurate Optical Material Parameter Determination with THz Time-Domain Spectroscopy*, Opt. Express, Vol. 15, Issue 7, Pp. 4335-4350 **15**, 4335 (2007).

[3] U. Tayvah, J. A. Spies, J. Neu, and C. A. Schmuttenmaer, *Nelly: A User-Friendly and Open-Source Implementation of Tree-Based Complex Refractive Index Analysis for Terahertz Spectroscopy*, Anal. Chem. **93**, 11243 (2021).

[4] Pochi Yeh, *Optical Waves in Layered Media* (Wiley-Interscience, 2005).

[5] A. Roggenbuck, H. Schmitz, A. Deninger, I. C. Mayorga, J. Hemberger, R. Güsten, and M. Grüninger, *Coherent Broadband Continuous-Wave Terahertz Spectroscopy on Solid-State Samples*, New J. Phys. **12**, 043017 (2010).

[6] S. Hazra, R. Damari, A. Golombek, E. Flaxer, T. Schwartz, and S. Fleischer, *Enhanced Transmission at the Zeroth-Order Mode of a Terahertz Fabry–Perot Cavity*, ACS Omega **9**, 3000 (2024).

[7] G. P. Papari, A. L. Pellegrino, G. Malandrino, and A. Andreone, *Sensing Enhancement of a Fabry-Perot THz Cavity Using Switchable VO 2 Mirrors*, Opt. Express **30**, 19402 (2022).

[8] J. Ji, A. Mousawi, J. Scheers, A. J. Jumaah, S. Al-Daffaie, and J. G. Rivas, *Exploring Phase Topology and Group Index Modifications of THz Vibro-Polaritons in Metasurfaces*, ACS Photonics **12**, 2203 (2025).

[9] P. A. Thomas, W. J. Tan, H. A. Fernandez, and W. L. Barnes, *A New Signature for Strong Light–Matter Coupling Using Spectroscopic Ellipsometry*, Nano Lett. **20**, 6412 (2020).

[10] I. Dolado et al., *Remote Near-Field Spectroscopy of Vibrational Strong Coupling between Organic Molecules and Phononic Nanoresonators*, Nat. Commun. **13**, 6850 (2022).
